# A litmus test for classifying recognition mechanisms of transiently binding proteins

**DOI:** 10.1038/s41467-022-31374-5

**Published:** 2022-07-01

**Authors:** Kalyan S. Chakrabarti, Simon Olsson, Supriya Pratihar, Karin Giller, Kerstin Overkamp, Ko On Lee, Vytautas Gapsys, Kyoung-Seok Ryu, Bert L. de Groot, Frank Noé, Stefan Becker, Donghan Lee, Thomas R. Weikl, Christian Griesinger

**Affiliations:** 1 Division of Sciences, Krea University, Sri City, India; 2 Department of NMR Based Structural Biology, Max Planck Institute for Multidisciplinary Sciences, Göttingen, Germany; 3 Department of Computer Science and Engineering, Chalmers University of Technology, Gothenburg, Sweden; 4 Department of Mathematics and Computer Science, Freie Universität Berlin, Berlin, Germany; 5 Research Center for Bioconvergence Analysis, Korea Basic Science Institute, Korea Basic Science Institute, Ochang-Eup, Cheongju-Si, South Korea; 6 Department of Theoretical and Computational Biophysics, Max Planck Institute for Multidisciplinary Sciences, Göttingen, Germany; 7 Department of Physics, Freie Universität Berlin, Berlin, Germany; 8 Department of Chemistry, Rice University, Houston, TX USA; 9 Department of Medicine, James Graham Brown Cancer Center, University of Louisville, Louisville, KY USA; 10 Department of Biomolecular Systems, Max Planck Institute of Colloids and Interfaces, Potsdam, Germany

**Keywords:** Solution-state NMR, Molecular modelling, Ubiquitins, Computational models

## Abstract

Partner recognition in protein binding is critical for all biological functions, and yet, delineating its mechanism is challenging, especially when recognition happens within microseconds. We present a theoretical and experimental framework based on straight-forward nuclear magnetic resonance relaxation dispersion measurements to investigate protein binding mechanisms on sub-millisecond timescales, which are beyond the reach of standard rapid-mixing experiments. This framework predicts that conformational selection prevails on ubiquitin’s paradigmatic interaction with an SH3 (Src-homology 3) domain. By contrast, the SH3 domain recognizes ubiquitin in a two-state binding process. Subsequent molecular dynamics simulations and Markov state modeling reveal that the ubiquitin conformation selected for binding exhibits a characteristically extended C-terminus. Our framework is robust and expandable for implementation in other binding scenarios with the potential to show that conformational selection might be the design principle of the hubs in protein interaction networks.

## Abstract (web summary)

The authors provide a litmus test for the recognition mechanism of transiently binding proteins based on nuclear magnetic resonance and find a conformational selection binding mechanism through concentration-dependent kinetics of ubiquitin and SH3.

## Introduction

Protein-ligand or protein-protein interactions underpin biological control mechanisms, and a detailed kinetic understanding of the interactions with atomic resolution is necessary to develop drug molecules. The role of molecular motion in these interactions is a very long-standing question, especially in the regime of fast kinetics^1^. Therefore, characterization of protein-ligand or protein-protein (protein-partner) interactions has been of interest for a long time, and the development of new experimental methods is crucial^2–14^. This line of research is critical to disentangle almost all molecular recognition in a cell^15^, including understanding the binding mechanism in terms of two-state binding vs. three-state binding via conformational selection or induced fit^16–18^.

**Fig. 1:**
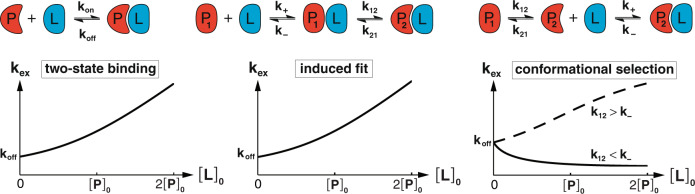
Concentration dependence of exchange rates in two-state and three-state binding mechanisms. Exchange rate *k*_ex_ for residues of the protein *P* as a function of the total concentration [*L*]_0_ of the ligand *L* in two-state binding, in three-state binding via induced fit, and in three-state binding via conformational selection. In the three-state binding models, the protein exhibits two conformations P_1_ and P_2_. In two-state binding and induced fit, *k*_ex_ increases with [*L*]_0_. In conformational selection, *k*_ex_ decreases with increasing [*L*]_0_ if the conformational excitation rate *k*_12_ is smaller than the unbinding rate *k*_−_ and increases with [*L*]_0_ if *k*_12_ is larger than *k*_−_. The exchange rate for two-state binding (TS) is ${k}_{{{{{{{{\rm{ex}}}}}}}}}^{{{{{{{{\rm{TS}}}}}}}}}={k}_{{{{{{{{\rm{on}}}}}}}}}{[{{{L}}}]}_{{{{{{{{\rm{eq}}}}}}}}}+{k}_{{{{{{{{\rm{off}}}}}}}}}$, and the exchange rates for induced fit (IF) and conformational selection (CS) are the dominant relaxation rates ${k}_{{{{{{{{\rm{ex}}}}}}}}}^{{{{{{{{\rm{IF}}}}}}}}}=\frac{1}{2}(S-\sqrt{{S}^{2}-4({k}_{+}{[{{{L}}}]}_{{{{{{{{\rm{eq}}}}}}}}}({k}_{12}+{k}_{21})+{k}_{21}{k}_{-})})$ and ${k}_{{{{{{{{\rm{ex}}}}}}}}}^{{{{{{{{\rm{CS}}}}}}}}}=\frac{1}{2}(S-\sqrt{{S}^{2}-4({k}_{12}({k}_{+}{[{{{L}}}]}_{{{{{{{{\rm{eq}}}}}}}}}+{k}_{-})+{k}_{-}{k}_{21})})$ of these three-state mechanisms with *S* = *k*_12_ + *k*_21_ + *k*_+_[*L*]_eq_ + *k*_−_. The equilibrium concentration [*L*]_eq_ of the unbound ligand is related to the total concentrations [*P*]_0_ and [*L*]_0_ of protein and ligand via ${[{{{L}}}]}_{{{{{{{{\rm{eq}}}}}}}}}=\frac{1}{2}({[{{{L}}}]}_{0}-{[{{{P}}}]}_{0}-{K}_{{{{{\rm{d}}}}}}+\sqrt{{({[{{{L}}}]}_{0}-{[{{{P}}}]}_{0}+{K}_{{{{{\rm{d}}}}}})}^{2}+4{[{{{P}}}]}_{0}{K}_{{{{{\rm{d}}}}}}})$ with the dissociation constants ${K}_{{{{{\rm{d}}}}}}^{{{{{{{{\rm{TS}}}}}}}}}={k}_{{{{{{{{\rm{off}}}}}}}}}/{k}_{{{{{{{{\rm{on}}}}}}}}}$, ${K}_{{{{{\rm{d}}}}}}^{{{{{{{{\rm{CS}}}}}}}}}={k}_{-}({k}_{12}+{k}_{21})/({k}_{+}{k}_{12})$, and ${K}_{{{{{\rm{d}}}}}}^{{{{{{{{\rm{IF}}}}}}}}}={k}_{-}{k}_{21}/({k}_{+}({k}_{21}+{k}_{12}))$ of the binding mechanisms (see Supplementary Methods for details).

In conformational selection^19,20^ and induced fit^21^, a conformational change occurs either prior to or after binding (Fig. 1). Prominent examples for induced fit include conformational changes from an open to a closed protein conformation after ligand binding^22^. Here, induced fit as binding mechanism can be directly deduced from protein structures if the entrance to the ligand-binding site is sterically blocked in the closed conformation of the bound form^22^. Other prominent examples for induced fit are protein systems with two bound forms of disordered fragments observed in nuclear magnetic resonance (NMR) experiments^23,24^. In the pKID/KIX system, the exchange between the free form and the bound forms is slow on the chemical shift timescale, which results in distinct peaks of these forms in NMR spectra^23^. Three-state fitting of NMR relaxation dispersion data and characteristic chemical shift changes during titration then directly evidence the existence of a binding mechanism with three states, whose structural identity can also be derived from chemical shift changes. Conformational selection in protein binding has been pioneered in NMR experiments that demonstrated conformational exchanges in the free protein form that are comparable to structural changes between the free and bound forms^7,25^. Relaxation-dispersion NMR methods to characterize low-populated conformations in free protein forms have been recently extended^26^ using paramagnetically induced pseudocontact shifts to increase the chemical shift range between different conformations^27^. But as a binding mechanism, conformational selection requires the additional kinetic proof that excited states observed e.g. in NMR experiments of the free form are on-pathway in the binding reaction. For protein binding reactions with relaxation times of milliseconds to seconds, such a kinetic proof can be provided by stopped-flow mixing experiments^18,28,29^. However, a general approach to investigate protein binding mechanisms is missing on sub-millisecond time scales where stopped flow is too slow or where the exchange between the free and bound protein forms is fast on the NMR chemical shift timescale under all stoichiometric conditions.

Here, we report the development of a theoretical and experimental framework for investigating protein-partner interaction with recognition kinetics down to tens of microseconds with atomistic detail. The framework can be applied to any binding regime but is particularly insightful for weak, transient binding with off-rates *k*_off_ larger than 1000 s^−1^. The kinetics of ligand binding are measured using high-power relaxation dispersion experiments, which have been shown to reveal kinetics down to single digit microseconds in individual proteins^30^. High-power relaxation dispersion is uniquely advantageous for measurement of both slow (<1000 s^−1^) and fast (up to ~ 37,000 s^−1^) kinetics in comparison to *R*_1*ρ*_ or off-resonance *R*_1*ρ*_ experiments regarding both experimental setup and data analysis^30^. We apply our framework to analyze the binding of the paradigmatic protein ubiquitin^7^ to its partner protein, the SH3c domain of CIN85. The interaction of ubiquitin and the SH3c domain is weak and transient (with dissociation constant *K*_d_ = 370 ± 15 μM from NMR titrations), akin to many other biologically important interactions. We show with the measurement of concentration-dependent kinetics using relaxation dispersion in the fast-exchange regime (Supplementary Fig. 1) that three-state binding via conformational selection dominates the kinetics of the binding on the side of ubiquitin. For the partner protein SH3c, we find consistence with two-state binding, in agreement with three-state conformational selection on the ubiquitin side. This concentration-dependent relaxation dispersion measurement and fitting procedure constitutes a litmus test for the recognition mechanism. In a subsequent step, we use molecular dynamics simulations and Markov state modeling^31–41^ to identify the ubiquitin conformation selected for binding. This binding-competent ubiquitin conformation exhibits a characteristically extended C-terminus.

Ubiquitin is a hub of the cellular interaction network. At the same time, CIN85 is an adapter molecule that controls the spatial and temporal assembly of multi-protein complexes by its three SH3 domains that bind other partners^42^. Thus, the interaction of ubiquitin with the third SH3 domain of CIN85 (SH3c) is the natural choice for testing our framework. Besides, given that the precise interaction of ubiquitin with many partners is a hallmark of cell function, it makes one wonder if there is a conformational selection mechanism in ubiquitin, where a minor “binding-compatible” conformation binds the partner specifically. Previous work has shown that free ubiquitin consists of an ensemble of conformations, including the “bound-like” conformations seen in ubiquitin complexes, thus supporting conformational selection as the binding mechanism^7^. Subsequent experimental and computational work has delineated the dynamic modes of ubiquitin in granular details^43^. Here we show how our expandable theoretical and experimental framework brings together the different internal dynamics and explains this paradigmatic protein-partner interaction.

## Results and discussion

### Measuring ligand-concentration dependent high-power relaxation dispersion enables distinguishing between binding mechanisms

**Fig. 2:**
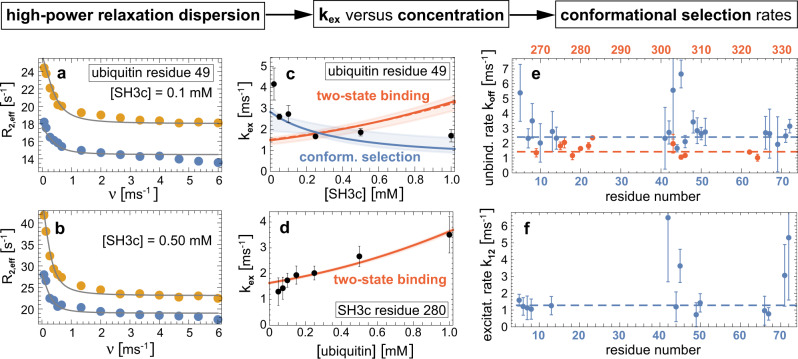
From relaxation dispersion NMR data to binding mechanisms. **a**, **b** Effective transverse relaxation rate *R*_2,eff_ versus nutation frequency *ν* of the applied transverse field measured by ^15^N relaxation dispersion for the amide of the ubiquitin residue 49 in the presence of 0.1 mM and 0.5 mM of SH3c. The blue and yellow data points result from measurements at the two ^15^N resonance frequencies 60.795 MHz and 96.313 MHz. The gray lines represent fits in the fast-exchange regime to determine the exchange rate *k*_ex_ (“Methods”). **c** The obtained exchange rates *k*_ex_ of the ubiquitin residue 49 (black data points) decrease with increasing SH3c concentration, which indicates conformational selection. The blue and red lines with shaded error regions result from fits of the *k*_ex_ equations of the two-state and conformational-selection binding mechanism (Fig. 1, Supplementary Methods). **d** For the amide of the SH3c residue 280, the exchange rate *k*_ex_ increases with the ubiquitin concentration and can be well fitted with the *k*_ex_ equation of two-state binding. **e** Unbinding rates *k*_off_ obtained from fits with the conformational-selection model for ubiquitin residues (blue data points) and from fits with the two-state binding model for SH3c residues (red data points, Supplementary Figs. 3 and 4). **f** Conformational excitation rate *k*_12_ from conformational-selection fits of ubiquitin residues (Supplementary Fig. 3). Global and residue-specific uncertainties in the *R*_2,eff_ values were estimated as described in Methods. The larger one of these two uncertainty estimates for each data is reported (smaller than the plot markers). The error bars in (**c**–**f**) represent standard errors of data fits (“Methods” and Supplementary Methods). Source data are provided as a Source Data file.

We characterized both proteins starting with ubiquitin since we had previously demonstrated that binding-competent conformations exist in the ubiquitin ensemble in the absence of binding partners^7^. The presence of binding-competent conformations is a necessary but not sufficient condition for conformational selection as the question is essentially about binding kinetics^16,44^. We previously determined fast conformational transitions with exchange rates *k*_ex_ larger than about 20,000 s^−1^ in free ubiquitin using relaxation dispersion^43^. NMR titration indicates also fast exchange between the free and bound forms of ubiquitin and SH3c, because we observed only one cross peak for all ratios of ubiquitin and SH3c (Supplementary Fig. 1), and because the intensity of this peak decreased monotonously with increasing formation of the complex (Supplementary Fig. 1e, i). Thus, we determined the concentration-dependent exchange rate *k*_ex_ of the complex formation at different partner concentrations as in our previous experiments on free ubiquitin by fitting the relaxation rates *R*_2,eff_ with the fast-exchange Luz-Meiboom equation^45^ (Fig. 2a, b and “Methods”). To gain insight on the binding mechanism from these experimentally determined *k*_ex_ values, we have developed analytical equations for the variation of *k*_ex_ with varying partner concentration, for two-state binding without (kinetically) relevant conformational change during binding, and for three-state binding with a conformational change prior to the binding step (conformational selection), or after binding (induced fit) (Fig. 1). These equations for *k*_ex_ hold at all concentrations of the proteins, in contrast to related equations for the dominant relaxation rate *k*_obs_ of stopped-flow mixing experiments derived under the ‘pseudo-first assumption’ of an excess concentration of one of the binding partners (Supplementary Methods). The analytical equations guided us in selecting conditions for the measurement of kinetic parameters for the ubiquitin-SH3c system, starting with a ratio of 1 (SH3c) to 50 (ubiquitin) and increasing the concentration of SH3c to 1:1. This wide sub-stoichiometric range of ubiquitin-SH3c ratios allows to identify the slope and curvature of *k*_ex_ as a function of the SH3c concentration [*L*]_0_ and to determine the unbinding rate *k*_off_ in the limit [*L*]_0_ to 0 (Fig. 1). The measurements reveal 22 ubiquitin residue positions with *k*_ex_ values that are clearly smaller than in free ubiquitin and, thus, reflect the exchange between the SH3c-bound and unbound state of ubiquitin (Supplementary Methods, Supplementary Table 2, and Supplementary Fig. 2). The exchange rate *k*_ex_ decreases with increasing total concentration of the binding partner SH3c at the large majority of the 22 residue positions (Fig. 2c and Supplementary Fig. 3), which signifies conformational selection and excludes two-state binding and induced fit (Fig. 1). The rate parameters for the conformational-selection model obtained from fitting of the concentration-dependent *k*_ex_ data at the 22 residues positions are overall consistent (Fig. 2e, f) and support conformational selection of a low-populated, excited ubiquitin conformation prior to binding to SH3c. Weighted averaging of the fitted rate parameters leads to the conformational excitation rate *k*_12_ = 1280 ± 170 s^−1^ and to the unbinding rate *k*_off_ = *k*_−_ = 2420 ± 140 s^−1^ (dashed blue lines in Fig. 2e, f). The population *k*_12_/(*k*_12_ + *k*_21_) of the excited unbound ubiquitin conformation is not larger than about 6.5% because the rate *k*_12_ + *k*_21_ for the conformational exchange in free ubiquitin is not smaller than about 20,000 s^−1^ according to previous measurements^43^.

Measurements on the side of SH3c reveal 12 residue positions with *k*_ex_ values affected by ubiquitin as binding partner (Supplementary Methods, Supplementary Table 3, and Supplementary Fig. 2). The *k*_ex_ curves at these 12 residue positions are consistent with two-state binding, in agreement with a conformational-selection three-state binding mechanism for ubiquitin, in which SH3c has two states (Fig. 2d and Supplementary Fig. 4). Weighted averaging of the single fit parameter *k*_off_ leads to *k*_off_ = 1.43 ± 0.04 ms^−1^ (or 1430 ± 40 s^−1^), which is close to the unbinding rate *k*_off_ obtained from the fits of the conformational-selection model on the side of ubiquitin.

### Markov modeling identifies a ubiquitin C-terminal mode as conformational-selection mode

To identify the ubiquitin conformation selected for binding, we carried out approximately 1.68 ms of molecular dynamics simulations and used these to build a Markov state model (MSM)^31,32^ that describes the conformational dynamics during binding as a kinetic network of metastable states (see Methods). The most stable state of the MSM is a structurally diverse, bound state that encompasses two published ubiquitin:SH3c models (PDB 2K6D and 2JT4)^46,47^. A comparison to previously reported distances derived from paramagnetic relaxation enhancement (PRE) measurements^46^ indicates that this bound state of our MSM recapitulates the experimental bound state well (Supplementary Fig. 5). Based on this bound state and an unbound state in which the distance of ubiquitin and SH3c is larger than 1 nm, we employ transition path theory^48–50^ to compute a committor probability, or binding probability, *p*_bind_ that quantifies the progress along the binding transition paths of the MSM (see “Methods”). We use adaptive sampling to access intermediate and unbound states with *p*_bind_ < 1^41^. Overall, the MSM resolves the reversible binding process of ubiquitin and SH3c in atomic detail and predicts a dissociation constant of binding that agrees with the experimental value within the statistical uncertainty (“Methods”). Markov state modeling and molecular dynamics simulations have been previously used to investigate the conformational changes of proteins during binding to small ligands^38,39,51–53^ and the binding-induced folding of disordered peptides^40,54–57^.

A peptide-flip motion between “in” and “out” conformations of ubiquitin emerged as a slow motion in earlier work^43^ and, thus, as possible candidate of a conformational-selection mode. However, the previously described mutant G53A that locks ubiquitin almost fully into the “out” conformation along the peptide-flip motion and the novel G53(D)T mutant (chemically synthesized with (D)-Threonine at position 53 and E24 ^15^N labeled as a reporter) that locks ubiquitin almost fully into the “in” conformation (see Methods) do not have a large effect on the dissociation constant *K*_d_ of ubiquitin and SH3c, with ${K}_{{{{{\rm{d}}}}}}^{{{{{{{{\rm{G53(D)T}}}}}}}}}=374\pm 48$ μM, ${K}_{{{{{\rm{d}}}}}}^{{{{{{{{\rm{G53A}}}}}}}}}=537\pm 28$ μM (Supplementary Fig. 6). These observations suggest that ubiquitin can bind SH3c in both the “in” and “out” conformation, and that the population-shift of the peptide-flip motion during binding to SH3c is rather small. The population shift can be calculated from the ratio of the dissociation constants for the “in” and “out” conformations and, thus, from the ratio of ${K}_{{{{{\rm{d}}}}}}^{{{{{{{{\rm{G53(D)T}}}}}}}}}$ and ${K}_{{{{{\rm{d}}}}}}^{{{{{{{{\rm{G53A}}}}}}}}}$ (Supplementary Methods). The binding-induced population shift of the peptide-flip motion in our Markov state model is also small, in agreement with the mutational data. As in previous molecular dynamics simulations^43^, the peptide-flip motion in our simulations is accelerated compared to the experiments. Similar to the peptide flip, the population shift of the pincer mode of ubiquitin^58^ during binding to SH3c is rather small in the MSM (Supplementary Methods).

**Fig. 3:**
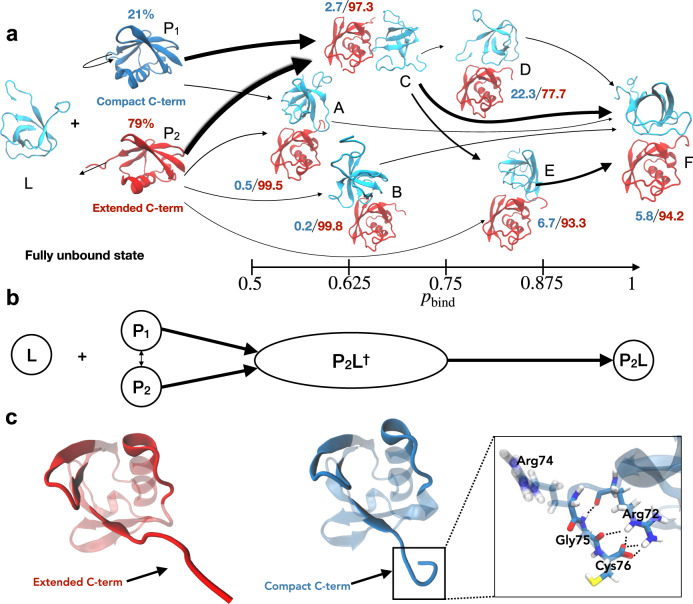
Ubiquitin-SH3c binding mechanism in the Markov state model. **a** Reactive flux along the dominant binding pathways, minor flux states are omitted from visual representation for clarity. The magnitude of the flux along different binding pathways is represented qualitatively by the width of the arrows that interconnect the states. Ubiquitin is shown in red in Markov states in which it predominantly adopts the extended C-terminal conformation P_2_. In the unbound state with compact C-terminal conformation P_1_, ubiquitin is shown in blue. SH3c as ligand L is shown in cyan. The relative probabilities of the compact and extended C-terminal conformation in the different states are indicated in blue and red. The probabilities *p*_bind_ of the Markov states for reaching the native bound state prior to the fully unbound state are given at the bottom. **b** Coarse view of the binding mechanism with the unbound ubiquitin states P_1_ and P_2_ and the binding transition state P_2_L^†^ and bound state P_2_L in which ubiquitin predominantly adopts the conformation P_2_ with extended C-terminus. The binding transition state P_2_L^†^ includes all Markov states with intermediate binding probabilities 0.45 < *p*_bind_ < 0.75. **c** Representative ubiquitin structures with extended and compact C-terminus. Interactions that stabilize the compact C-terminal conformation are illustrated at the right.

**Fig. 4:**
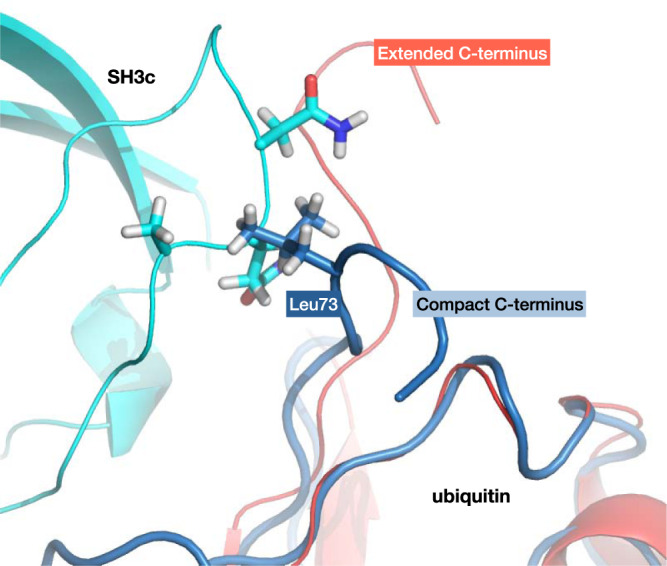
Compact C-terminal ubiquitin conformation sterically obstructs binding to SH3c via the ubiquitin Leu73 sidechain. Superposition of compact C-terminal ubiquitin conformations from simulations (blue) on the experimental ubiquitin:SH3c model (pdb: 2k6d, red and cyan) show steric clashes between ubiquitin Leu73 and the SH3c backbone.

Besides the peptide-flip mode, an independent and similarly slow motion in our simulations and Markov modeling involves the flexible C-terminal tail of ubiquitin. In free ubiquitin, we observe two distinct compact and extended conformations of the C-terminal tail, which we define via time-lagged independent component analysis^59,60^ of the C-terminal backbone torsion angles of ubiquitin, considering only the unbound states with *p*_bind_ = 0 (“Methods”). Our Markov model constructed from 1.68 ms of binding simulations indicates that the population of the compact C-terminal conformation is strongly reduced during binding, and that this population reduction occurs prior to the transition state of binding, which is a clear signature of conformational selection. Fig. 3a illustrates the reactive flux between the dominant coarse-grained states of our MSM in binding direction. Along the binding pathways, the population of the compact C-terminal conformation diminishes from 21% (confidence interval (CI): 17–25%) for the compact, unbound state P_1_ to 2.4% (CI: 1.7–3.4%) in the transition-state ensemble, which is composed of the states A, B, and C with intermediate binding probability 0.45 < *p*_bind_ < 0.75, and remains low in the bound state F with a population value of 5.8% (CI: 4.2–7.8%). The vanishing population of the compact conformation in the transition state implies that productive binding events, across the transition state, are not possible in this conformation. Unlike the extended C-terminal conformation, the compact conformation sterically obstructs binding of SH3c to ubiquitin (Fig. 4). Consequently, the extended conformation of the C-terminus likely is the sought-after ubiquitin conformation selected for binding. Based on our fits of the *k*_ex_ data, we expect a more drastic shift in populations for the conformational-selection mode, i.e., a larger population of the compact C-terminal conformation in the unbound state. However, the discrepancy we observe between experiment and modeling is within systematic errors in state-of-the-art molecular dynamics force-fields^61,62^ that were the basis of the MSM. Relative populations of alternative conformations are notoriously difficult to estimate from molecular dynamics simulations, because systematic errors of few kJ mol^−1^ can lead to large deviations in populations.

In summary, we introduce a litmus-test-like theoretical and experimental framework to identify conformational selection of transiently binding proteins on sub-millisecond timescales that are beyond the reach of standard stopped-flow mixing experiments or NMR methods relying on intermediate or slow exchange between bound and unbound protein forms. Our framework extends the time resolution in protein binding experiments in a way that is comparable to the timescale extension provided by temperature-jump experiments of protein folding relative to stopped-flow mixing experiments^63,64^. We expect that this framework will be applicable for many transient complexes. For the paradigmatic ubiquitin-SH3c complex, we identify conformational selection of ubiquitin, which agrees with the two-state recognition mechanism observed for the binding partner SH3c. In a complementary computational approach that involves molecular dynamics simulations and Markov modeling, we find that the ubiquitin conformation selected for binding exhibits a characteristically extended C-terminus. This framework makes future explorations possible to test the hypothesis that hub proteins such as ubiquitin utilize conformational selection as an evolutionary mechanism to be more adaptable.

## Methods

### Expression, purification and NMR sample preparation of the SH3c domain of *human* CIN85

The ^15^N-labeled SH3c domain of hCIN85 was recombinantly produced in Toronto minimal medium with ^15^N-NH_4_Cl (Sigma Aldrich) as nitrogen source according to a published protocol^42^. Briefly, a fragment of CIN85 comprising amino acids 263-333 was expressed in the bacterial strain BL21(DE3)Star (Invitrogen) as fusion protein with N-terminal His_7_-tag. After purification on a Ni-NTA Protino^TM^ metal affinity column (Macherey-Nagel, Germany) the His_7_-tag was cleaved off with TEV-protease and removed by a second Ni-NTA Protino^TM^ column purification step. The SH3c (this construct is referred to as SH3c in the main manuscript) domain was eluted in the flow-through and further purified by gel-filtration on a Superdex 75/16-60 column (GE Healthcare). The sample was dialyzed against NMR buffer (20 mM sodium phosphate, pH 6.5, 100 mM NaCl, 10 mM TCEP, 0.05% (w/v) NaN_3_) and the final concentration was adjusted to 2 mM.

### Chemical synthesis, folding, purification and NMR sample preparation of ^15^N-Glu_24_-labeled D-Thr_53_-ubiquitin

**Fig. 5:**
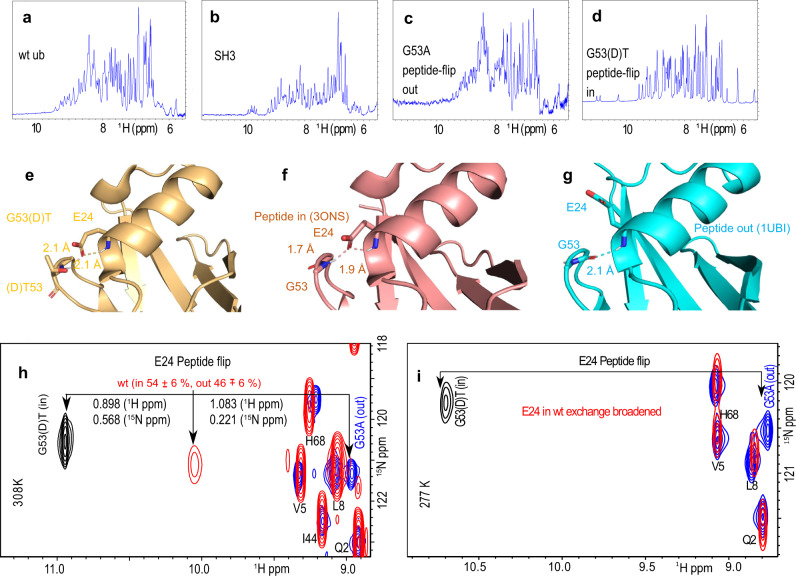
NMR spectroscopy characterization of SH3c and ubiquitin wt and mutants G53(D)T and G53A. **a**–**d** 1H NMR spectra of the amide region showing for all proteins a range of chemical shifts up to or more than 9 ppm, indicating that the two mutants of ubiquitin as well as the wt and the construct of SH3c are well-folded in the buffer condition used for the experiments. **e**–**g** crystal structures of the G53(D)T mutant as well as the two crystal structures of wt ubiquitin showing the “in” conformation of the peptide bond (**f**) and the “out” conformation (**g**). The G53(D)T (**e**) mutant of ubiquitin (golden ribbon) has a similar poise of the G53 peptide-bond and the side-chain of E24 as the wild-type ubiquitin in the “in” conformation (**f**, magenta ribbon, PDB: 3ONS). The side-chain of the (D)T53 is shown in stick representation. The dihedral angles for (D)T53 (*ϕ* : 109.7°; *ψ* : 18.6°) show that the molecules is locked into the peptide-flip “in” conformation. For comparison, the dihedral angles of G53 in the “in” conformation are *ϕ* : 98.6°, and *ψ* : −25.6° (PDB: 3ONS) and “out” conformation along the peptide-flip mode, are *ϕ* : −82.9°, and *ψ* : −8.9° (PDB: 1UBI). HSQC spectra of the wt, the G53(D)T and G53A mutant of ubiquitin at 308K (**h**) and 277 K (**i**). The black arrows indicate the positions of the NH resonance of E24. The G53(D)T and G53A mutants were designed to redistribute the populations to “in” and “out” conformations along the peptide-flip mode, respectively.

Synthetic ^15^N-Glu_24_-labeled D-Thr_53_-ubiquitin was produced by Fmoc protection-based^65^ linear solid-phase peptide synthesis (SPPS) with an automated microwave synthesizer (Liberty 1, CEM), similar to a published protocol for high-yield synthesis of ubiquitin^66^ (0.1 mM scale, fivefold excess of amino acid for coupling, capping was done with 20% acetic acid anhydride). Briefly, synthesis was performed on an Fmoc-Gly preloaded Wang resin^67^ (Novabiochem). Couplings of the protected amino acids (Novabiochem) were performed with HBTU/HOBT/DIEA reagent mix^68^ (Merck), except for ^15^N-labeled Fmoc-Glu(OtBu)-OH (Sigma Aldrich) that was coupled overnight at position 24 using HATU/DIEA reagent mix^69^ (Merck). From position 24 onward only half the resin was reacted. From residue 52 onward no microwave irradiation was used to prevent aspartimide formation. The Fmoc-D-Thr-OH amino acid was incorporated at position 53. After deprotection with 20% piperidine and cleavage from the resin and lyophilization, the raw peptide (220 mg) was dissolved at 10 mg/ml in DMSO and refolded at room temperature by dropwise dilution into buffer A (50 mM acetic acid, pH 4.5) to a final DMSO concentration of 2% (v/v). The refolded protein solution was sequentially purified on a 5 ml HiPrep SP XL cation exchange column (GE Healthcare) and on a Mono S HR5-5 cation exchange column (GE Healthcare). The correct mass of the purified protein was verified by LC-MS (column: XSelect Peptide CSH C18 XP column, 2.5 μm, 4.6 × 100 mm, 130 Å, Waters; Acquity Arc System, Waters with SQD2-Mass-Detector (Single Quadrupol)) after each column purification step. All fractions from both columns were investigated by LC–MS (molecular weight measured: 8612 Da, expected: 8610 Da) and the purest fractions were pooled. For preparing the NMR sample the protein was dialyzed overnight against 20 mM sodium phosphate, pH 6.5, 100 m NaCl. After addition of 10 mM TCEP, 10% D2O (v/v) and 0.05% NaN_3_ (w/v), the final protein concentration was adjusted to 2 mM. The volume of the NMR sample following the dialysis and concentration steps was 350 μL (5.95 mg in total). The spectra showed that the protein is folded (Fig. 5). All other chemicals were purchased from Merck, Sigma, Alfa Aesar and Multisyntech.

### Crystallization of D-Thr_53_-ubiquitin, data collection and structure determination

Protein from the NMR sample was also used for crystallization. Crystals were obtained at 20 °C by sitting drop vapor diffusion mixing 100 nL of protein solution with 100 nL of well solution (0.1 M Bis-Tris, pH 7.5, 20% polyethylene glycol monomethyl ether 2000, 50 mM CdCl_2_). For data collection crystals were soaked for 1 min in well buffer supplemented with 20% (v/v) glycerol. Data collection was performed at SLS Villigen, Switzerland (beamline PXII, Pilatus 6M detector). Data were processed with XDS^70^. Space group determination and statistical analysis (Supplementary Table 1) was performed with XPREP (Bruker AXS, Madison, Wisconsin, USA). The structure was solved at a resolution of 2.6 Å by molecular replacement with PHASER^71^ using the crystal structure of ubiquitin (PDB code: 1UBQ)^72^ as search model. Refinement (Supplementary Table 1) was performed with Refmac5^73^ alternating with manual model building in Coot^74^. The crystal structure has the same overall fold as the wild-type ubiquitin except the region near residue position 53 (Fig. 5).^75,76^. All mutants and wild-type ubiquitin were isotopically labeled, expressed and purified as described^77^.

### Kinetics of interconversion of free wt, G53A, and G53(D)T mutant

HSQC spectra were recorded for wt ubiquitin and its G53A and G53(D)T mutants. The chemical shifts of E24 amide are vastly different, by 1.981 ppm in the proton dimension due to the different chemical environment of the two mutants (Fig. 5). The wild-type ubiquitin is a mixture of the “in” and “out” conformations of the peptide-flip motion, the chemical shift of the proton and nitrogen being more or less in the middle. The wild-type resonance measured at 400 MHz is visible only at 308 K. At 277, it is exchange broadened beyond detection. The HSQC of G53(D)T was measured at 308K in a 900 MHz spectrometer. The HSQC of the G53A mutant at 308 K was measured at 700 MHz spectrometer. To calculate the populations of the “in” and “out” conformation in the wt, we used the weighted average of the proton and nitrogen chemical shifts of E24 of the two mutants. The error in the position of the weakest peak E24 in wt was calculated as 1/2⋅linewidth/signal-to-noise. The error in peak position was propagated to obtain the population error of ±6%. The E24 in the “in” and “out” peptide-flip G53A and G53(D)T mutants are visible at 277 K, indicating that there is no exchange for the mutants indicating that they are locked in the “in” or “out” conformations, respectively. Both the G53A and G53(D)T mutants, corresponding to the “out” and “in” conformations are measured in 800 MHz spectrometer.

### High-power relaxation dispersion of ubiquitin (with SH3c titrated in)

The high-power relaxation experiments were measured using the ^15^N based constant time E-CPMG experiment^30^ for quantifying micro-to-millisecond time-scale exchange process in ubiquitin in Bruker Avance 600 MHz and 950 MHz spectrometers fitted with cryoprobe-TCI (Neo console running Topspin 4.× (Bruker Biospin corporation)) at 277 K. The constant time (CT) CPMG delay is divided into two equal halves, sandwiching the U-element that ensures the equal contribution of anti-phase and in-phase relaxation to *R*_2,eff_ in all frequencies. In the E-CPMG experiment performed here, refocusing pulses are applied with strong *γB*_1_/2*π* (7143 Hz and 7407 Hz for ^15^N in the 950 MHz and 600 MHz spectrometers respectively) fields (corresponding to ^15^N hard pulses) for all refocusing frequencies, thus reducing any off-resonance effects that can affect the measurement of *R*_2,eff_^30^. The *R*_2,eff_ values were measured at CPMG frequencies (*ν*_CPMG_) of 66.7, 133, 267, 400, 533, 667, 1333, 2000, 2667, 3333, 4000, 4667, 5333, and 6000 Hz (Supplementary Figs. 7–12).

The constant volume of 200 μL of NMR samples was put inside 3 mm tubes (Hilgenberg GmbH) in 20 mM sodium phosphate buffer, pH 6.5, containing 100 mM NaCl, 10mM TCEP, 0.05% (w/v) sodium azide, and 10% D_2_O. In all experiments the ubiquitin (^15^N labeled) concentration was 1 mM. The SH3c (unlabeled) concentration was varied from 0, 0.02, 0.05, 0.1, 0.25, 0.5 mM up to 1 mM. The probe temperature was calibrated using a digital thermometer and standard methanol sample.

The reference spectra were collected without the CPMG delay period (*τ*). The *R*_2,eff_ was calculated as

1 $${R}_{2,{{{{{{{\rm{eff}}}}}}}}}({\nu }_{{{{{{{{\rm{CPMG}}}}}}}}})=-1/T\log \left(I({\nu }_{{{{{{{{\rm{CPMG}}}}}}}}})/{I}_{0}\right)$$

where *ν*_CPMG_ is the effective frequency of the CPMG field, (*ν*_CPMG_ = 1/(4*τ*), where the time between the centers of consecutive 180^∘^ pulses is 2*τ*), *T* is the constant delay during which CPMG pulses were applied (60 ms), *I*_0_ is the intensity of the peak in reference experiment and *I*(*ν*) is the intensity of the peak at that particular CPMG frequency. The CPMG delay (60 ms) was chosen such that the residual intensity was approximately 50% of maximum intensity. The experiment was performed with 3 s recycle delay between increments using 12 different refocusing field strengths between 0 and 6000 Hz collected in scrambled and interleaved manner with 1024 (^1^H) and 130 (^15^N) complex points, respectively. For each increment, 16 transients were measured following the Echo-AntiEcho scheme for signal averaging. There is a heat compensation block in the middle of the recycle delay to dump the extra CPMG cycles so that the total number of CPMG 180^∘^ refocusing pulses at fixed *B*_1_ field strength is identical during the individual scans. The E-CPMG experiments took 3 days to complete, and standard ^1^H, ^15^N TROSY-HSQC spectra were collected before and after each experiment to monitor sample stability. A set of 5 non-exchanging residues were identified based on the criteria of lowest standard deviation between the *R*_2,eff_ values. The global uncertainty for the experimental data was calculated as the average of the standard deviations of the set of 5 residues^29^. The residue-specific uncertainties were calculated from measuring the deviation between *R*_2,eff_ values in repeat measurements at a suitable frequency (667 Hz). The largest of the global or residue-specific uncertainties is reported.

### High-power relaxation dispersion of SH3c (with ubiquitin titrated in)

The high-power relaxation dispersion on the ^15^N labeled SH3c were measured at 277 K in Bruker Avance-III 800 MHz spectrometer equipped with cryoprobe-TCI. The refocusing pulses were applied with *γ*B_1_/2*π ~* 5 kHz for ^15^N in an interleaved manner with 3 s recovery delay. The spectra were recorded with 1024 and 156 complex points in the direct and indirect dimensions, respectively. The NMR experiments were performed with the ^15^N-labeled CIN85-SH3 and unlabeled ubiquitin complex in 20 mM sodium phosphate buffer, pH 6.5, containing 100 mM NaCl, 10mM TCEP, 0.05% (w/v) sodium azide, and 10% D_2_O. In this experiment the SH3c (^15^N labeled) concentration was kept fixed at 1 mM, and the ubiquitin (unlabeled) concentration was varied from 0, 0.02, 0.05, 0.075, 0.1, 0.15, 0.25, 0.5 mM, up to 1 mM. The *R*_2,eff_ values were measured at the same frequencies as the previous experiment.

### Fast exchange of free and bound forms

Linear shifts of peaks in HSQC spectra upon titration indicate fast exchange of free and bound forms (Supplementary Fig. 1). Supplementary Fig. 1a shows a series of HSQC spectra upon titration of ubiquitin with SH3c, and Supplementary Fig. 1b shows the same for SH3c titrated with ubiquitin. The ratios between the two proteins ranged from 0 to 80% in the first case and from 0 to 78% in the second case. The amount of the bound complex was limited by the solubility of the proteins. The peaks shift linearly without indication of a third state. The linewidths both in the proton and nitrogen dimension increase with increasing concentration of the other component according to the increase of the effective molecular weight. No line broadening due to intermediate exchange is seen.

### Fitting of relaxation dispersion data with a two-state exchange model

We fitted the relaxation rates *R*_2,eff_ with the fast-exchange formula^2,45^

2 $${R}_{2,{{{{{{{\rm{eff}}}}}}}}}={R}_{2,0}({B}_{0})+\frac{{\psi }_{{{{{{{{\rm{ex}}}}}}}}}\,{{{{{{{{\rm{{B}}}}}}}_{0}}}}^{2}}{{k}_{{{{{{{{\rm{ex}}}}}}}}}}\left(1-\frac{4\nu }{{k}_{{{{{{{{\rm{ex}}}}}}}}}}\tanh \left[\frac{{k}_{{{{{{{{\rm{ex}}}}}}}}}}{4\nu }\right]\right)$$

On the ubiquitin side, the two NMR data sets for *R*_2,eff_ as a function of *ν* = 1/(4*τ*) at the two ^15^N resonance frequencies 60.795 MHz and 96.313 MHz (blue and yellow data points in Supplementary Figs. 7 to 12, respectively) were jointly fitted using the four fit parameters *R*_2,0_(60.795 MHz), *R*_2,0_(96.313 MHz), *ψ*_ex_, and *k*_ex_. The fit results for the two-state exchange rate *k*_ex_ at the different SH3c concentrations and ubiquitin residue positions are shown in Supplementary Table 2. We used the function NonlinearModelFit of Mathematica 11.3^78^ in these fits. The errors Δ*R*_2,eff_ of the data points were included as weights $1/{({{\Delta }}{R}_{2,{{{{{{{\rm{eff}}}}}}}}})}^{2}$ in the fitting, and the errors of the fit parameters were estimated from the fit residuals with the standard variance estimator function of NonlinearModelFit. Because of the typically smaller errors of the blue data points obtained at the ^15^N resonance frequency 60.795 MHz, the joint fits of the data at both resonance frequencies tend to be more faithful to these blue data, compared to the yellow data points obtained at the ^15^N resonance frequency 96.313 MHz (Supplementary Figs. 7–12).

On the SH3c side, the NMR data for *R*_2,eff_ as a function of *ν* at the ^15^N resonance frequency of 81.1 MHz were fitted with the three fit parameters *R*_2,0_(81.1 MHz), *ψ*_ex_, and *k*_ex_. The fit results for *k*_ex_ at the different ubiquitin concentrations and SH3c residue positions are shown in Supplementary Table 3. The errors were estimated from the fit residuals with the standard variance estimator function of NonlinearModelFit of Mathematica 11.3.

### Molecular dynamics simulations of ubiquitin-SH3c binding

We adopted the coordinates from the complex (PDB-file 2K6D) as a starting point to generate the topology for our simulation system. Several N- and C-terminal residues were missing in the SH3c chain when compared to the experimental construct. Consequently, amino acids GHMDSRT and DFEKE were added respectively to the N- and C-termini of the SH3c chain, using PyMOL. We performed all equilibration simulations using GROMACS 5.1.4^79^. We separated the ubiquitin and SH3c chains into independent simulation systems. These chains were independently solvated; we added Na^+^ and Cl^−^ ions to neutralize the simulation box, which was then energy minimized and equilibrated in the NpT ensemble for 100 ps. Finally, we equilibrated for five nanoseconds in the NVT ensemble at 330K with the Amber99SB-ILDN forcefield^80^. We used an integration time-step of 2 fs, kept the simulation box temperature using the Bussi-thermostat^81^, and treated long-range electrostatics using the Particle Mesh Ewald method. In the simulations of ubiquitin, we used a cubic box with side-length 6.55 nn that contained 8863 TIP3P water molecules, and protonated His68 at N*ϵ*. In the simualtions of SH3c, we used a cubic box with side-length 6.53 nm that contained 9084 TIP3P water molecules, 6 Na^+^ ions, and protonated His2 at N*ϵ*. Using PyMOL, we extract ten random configurations of the protein chains from the ubiquitin and SH3c equilibration simulations. We paired the ubiquitin and SH3c configurations together randomly, without replacement. Each pair of structures were placed randomly (non-overlapping) in a cubic box of side-length 10.0 nm.

Using GROMACS 5.1.4, we solvated each of ten starting orientations of ubiquitin and SH3c in 31,817 TIP3P water molecules, adding 66 Na^+^ and 60 Cl^−^ ions to a final concentration of 100 mM NaCl. The total system size is 98,995 atoms. We use the Amber99SB-ILDN forcefield, to energy minimize the simulation box, followed by equilibration in the NpT ensemble for 100 ps to a final box size of 10.0 nm^3^. We export the final system coordinates for the initialization of production simulations on graphics processing units (GPUs) in OpenMM 7.5^82^.

In our production simulations^82^, we used hydrogen-mass repartitioning with heavy protons (4 amu) and constrained all covalent bonds to enable a 4 fs integration time step. We used the Amber99SB-ILDN forcefield for the protein chains, and TIP3P for the water molecules. The Particle mesh Ewald method was used to treat electrostatic interactions beyond 0.9 nm. We integrated the system using a Langevin integrator with a friction constant of 1 ps^−1^ and thermostatting to 300 K. We performed 200 ps equilibration simulations of each of the starting configurations in the NVT ensemble, and observed no energy or temperature drift after a few ps.

We ran 1015 simulations in total, across five adaptive rounds, with approximately 200 concurrent simulations per round. The longest simulations were 4 μs, and 50% of all simulations were between 811 ns and 2 μs. We used an adaptive sampling strategy to encourage sampling of transitions between the bound and unbound states, while allowing a diverse set of associated states which may or may not lead to productive binding events. For every adaptive sampling round, we selected new starting points manually through visual inspection of representative conformational states identified in preliminary MSMs. We saved system coordinates every 0.2 ns, but strided into 1 ns steps for all subsequent analyses. We discarded the first nanosecond from each simulation as equilibration.

### Markov modeling

We built an MSM using features aiming to resolve the internal structural rearrangements in ubiquitin associated with its association to the SH3c domain using PyEMMA 2.5.7 and MDTraj 1.9.3^83–85^. Consequently, we selected a concise set of features, based upon available structural models of ubiquitin:SH3c complexes (PDB: 2K6D and 2JT4)^46,47^. We used two groups of features. The first group is composed of the shortest inter-residue distances between all residue pair combinations listed in Supplementary Table 4. We employed time-lagged independent component analysis^59,60^ (TICA) to reduce the dimension of these distances to six using a lag-time of 100 ns. These six dimensions represent native interface contacts in experimental models (PDB: 2K6D and 2JT4), which we combined with the shortest distance between ubiquitin and the N- and C-termini of SH3c (first 14 and last 10 residues) to a seven-dimensional space. The latter distance helps to resolve non-productive binding events. We clustered these radial features into 450 states, using k-means clustering.

The second group of features is composed of the cosines and sines of backbone torsions of the C-terminus of ubiquitin (residue 70-76). We employed TICA to reduce the dimension of these angular features to two using a lag-time of 50 ns. The first of these TICs is used to define a C-terminal mode, which undergoes a significant population shift during binding (Supplementary Methods). In the TICA analysis, we considered only unbound states with a ubiquitin and SH3c inter-chain distance of at least 1 nm. We clustered this 2D space into 12 cluster centers.

**Fig. 6:**
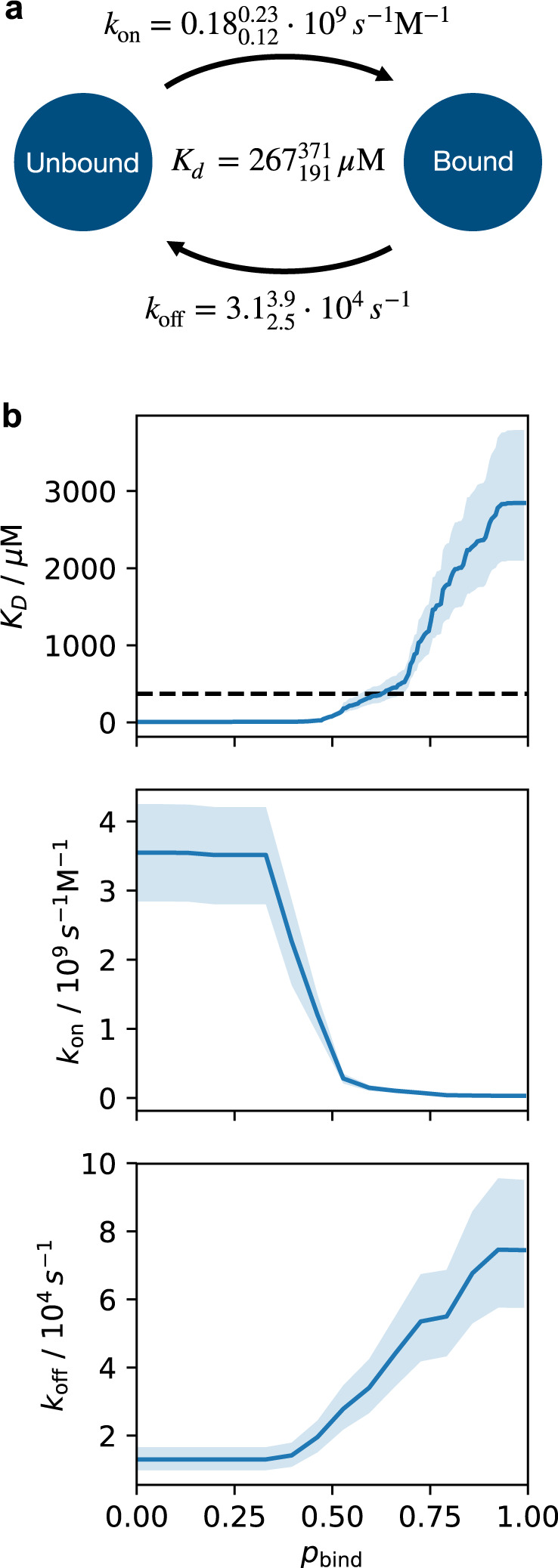
Binding kinetics in the MSM. **a** On-rate *k*_on_, off-rate *k*_off_, and dissociation constant *K*_*d*_ of ubiquitin and SH3c calculated from the MSM for the state threshold value ${p}_{{{{{{{{\rm{bind}}}}}}}}}^{* }=0.57$ at which the experimental value for *K*_*d*_ is obtained. Unbound/bound states of the MSM are defined as states with ${p}_{{{{{{{{\rm{bind}}}}}}}}}^{* }$ values smaller/larger than the state threshold value. Super and sub-scripts indicate a 95% confidence interval of the posterior distribution of the MSM transition matrix. **b** *K*_d_, *k*_on_, and *k*_off_, as a function of the state threshold value ${p}_{{{{{{{{\rm{bind}}}}}}}}}^{* }$. The threshold ${p}_{{{{{{{{\rm{bind}}}}}}}}}^{* }=0.57$ in (**a**) is located within a plausible transition state region. However, the entire range of predicted *K*_d_ values for different threshold choices is within the expected error of current state-of-the-art force field. The rates in subscript and superscript in (**a**) and the error regions in (**b**) represent 95% confidence intervals of the posterior distribution of Markov models.

Initially, we assigned bound configurations to one of the 450 states defined by the radial features and unbound configurations to one of the 12 states represented by the angular features. This procedure led to a total of 462 states. To resolve the peptide-flip mode, we further split all states into two separate states if a cluster center contains both in and out configurations. This step brought us to 924 states. To prune out weakly connected states and attenuate errors associated with our coarse system representation, we filtered our discrete state trajectories using a low-pass filter lag-time of 90 ns (Supplementary Methods). We selected features and split Markov states based on previously determined important structural features for intrinsic dynamics of ubiquitin as well as ubiquitin:SH3c binding. Our model does not resolve any internal degrees of freedom of the SH3c domain, and as such, the model only represents the encounter dynamics from the ubiquitin perspective. Following these steps we arrive at our molecular dynamics data mapped on to 607 Markov states, which we used for the posterior sampling of 5000 Markov state models at lag time 62 ns following the Bayesian formalism previously described^86^. We chose the lag time based on the implied timescales and on the populations of the 20 most highly populated states in the MSM as a function of lag-time (Supplementary Fig. 13). The implied timescales are the global relaxation timescales predicted by the MSM, and were computed via the Eigenvalues of the transition probability matrix^31^. Both implied timescales and state populations are stable (within model uncertainty) at the chosen lag time. A Chapman–Kolmogorov test (Supplementary Fig. 13) shows that the model is consistent with the simulation data on timescales longer than the lag time^31,50^. Repeated runs of this procedure led to slight variations in the final number of states due to the stochastic nature of k-means clustering. The binding dissociation constant *K*_d_ predicted by the MSM agrees with the experimentally determined value within the statistical uncertainty (Fig. 6).

To facilitate structural analysis, we coarse-grained the MSM by using Perron cluster–cluster analysis (PCCA) into 15 metastable states^87^. The unbound state is not metastable on the model lag-time. We consequently separate the unbound states into a separate set of states manually, as all Markov states with an average ubiquitin-SH3c distance of more than 1nm. We then group the unbound Markov states into extended and compact C-terminal states. This leaves us with 17 states in total, however, in the main text we only visualize the states involved with high net flux^40^ > 10^−7^ for visual clarity (Fig. 3). All the metastable states have substantial conformational flexibility which impedes detailed structural analysis of the individual states. We report key properties of the of the 17 states in Supplementary Table 5.

### Reporting summary

Further information on research design is available in the Nature Research Reporting Summary linked to this article.

## Supplementary information

Supplementary Information

Reporting Summary

## Data Availability

The NMR data of this study and the mass spectrometry data for the synthesis of the G53(D)Thr ubiquitin protein are available in the open research data repository Edmond at 10.17617/3.AVKYZC^88^. The molecular dynamics data of this study are available in the Edmond data repository at 10.17617/3.8o^89^. The structure factor file and the atomic coordinates of the G53(D)T mutant of ubiquitin have been deposited in the Protein Data Bank under the accession code 7OOJ. Previously published structures of ubiquitin-SH3c complexes used for a comparison to molecular dynamics conformations are available in the Protein Data Bank under the accession codes 2K6D and 2JT4. Source data are provided with this paper.

## Source data

Source Data

## Code availability

The code for the Markov model building of this study is available at https://github.com/olsson-group/litmus-test-paper.

## References

[1] Eigen M, Hammes GG, Kustin K (1960). Fast reactions of imidazole studied with relaxation spectrometry. J. Am. Chem. Soc..

[2] Mulder FA, Mittermaier A, Hon B, Dahlquist FW, Kay LE (2001). Studying excited states of proteins by NMR spectroscopy. Nat. Struct. Biol..

[3] Palmer, A. G., 3rd. NMR characterization of the dynamics of biomacromolecules. Chem. Rev. 104, 3623-40 (2004). 10.1021/cr030413t 15303831

[4] Mittermaier A, Kay LE (2006). New tools provide new insights in NMR studies of protein dynamics. Science.

[5] Boehr DD, Dyson HJ, Wright PE (2006). An NMR perspective on enzyme dynamics. Chem. Rev..

[6] Henzler-Wildman K, Kern D (2007). Dynamic personalities of proteins. Nature.

[7] Lange OF (2008). Recognition dynamics up to microseconds revealed from an RDC-derived ubiquitin ensemble in solution. Science.

[8] Loria JP, Berlow RB, Watt ED (2008). Characterization of enzyme motions by solution NMR relaxation dispersion. Acc. Chem. Res..

[9] Hammes GG, Chang Y-C, Oas TG (2009). Conformational selection or induced fit: a flux description of reaction mechanism. Proc. Natl. Acad. Sci. USA.

[10] Clore GM, Iwahara J (2009). Theory, practice, and applications of paramagnetic relaxation enhancement for the characterization of transient low-population states of biological macromolecules and their complexes. Chem. Rev..

[11] James LC (2003). Antibody multispecificity mediated by conformational diversity. Science.

[12] Bouvignies G (2011). Solution structure of a minor and transiently formed state of a T4 lysozyme mutant. Nature.

[13] Copeland RA (2015). The drug–target residence time model: a 10-year retrospective. Nat. Rev. Drug Discov..

[14] Mazal H, Haran G (2019). Single-molecule FRET methods to study the dynamics of proteins at work. Curr. Opin. Biomed. Eng..

[15] Alderson TR, Kay LE (2021). NMR spectroscopy captures the essential role of dynamics in regulating biomolecular function. Cell.

[16] Boehr DD, Nussinov R, Wright PE (2009). The role of dynamic conformational ensembles in biomolecular recognition. Nat. Chem. Biol..

[17] Paul F, Weikl TR (2016). How to distinguish conformational selection and induced fit based on chemical relaxation rates. PLoS Comput. Biol..

[18] Vogt AD, Cera ED (2012). Conformational selection or induced fit? A critical appraisal of the kinetic mechanism. Biochemistry.

[19] Monod J, Wyman J, Changeux JP (1965). On the nature of allosteric transitions: a plausible model. J. Mol. Biol..

[20] Ma B, Kumar S, Tsai CJ, Nussinov R (1999). Folding funnels and binding mechanisms. Protein Eng..

[21] Koshland DE (1958). Application of a theory of enzyme specificity to protein synthesis. Proc. Natl. Acad. Sci. USA.

[22] Sullivan SM, Holyoak T (2008). Enzymes with lid-gated active sites must operate by an induced fit mechanism instead of conformational selection. Proc. Natl Acad. Sci. USA.

[23] Sugase K, Dyson HJ, Wright PE (2007). Mechanism of coupled folding and binding of an intrinsically disordered protein. Nature.

[24] Schneider R (2015). Visualizing the molecular recognition trajectory of an intrinsically disordered protein using multinuclear relaxation dispersion nmr. J. Am. Chem. Soc..

[25] Boehr DD, McElheny D, Dyson HJ, Wright PE (2006). The dynamic energy landscape of dihydrofolate reductase catalysis. Science.

[26] Stiller, J. B. et al. Structure determination of high-energy states in a dynamic protein ensemble. *Nature*. 10.1038/s41586-022-04468-9 (2022). 10.1038/s41586-022-04468-9 PMC9126080 35236984

[27] Eichmüller C, Skrynnikov NR (2007). Observation of microsecond time-scale protein dynamics in the presence of ln^3+^ ions: application to the N-terminal domain of cardiac troponin C. J. Biomol. NMR.

[28] Vogt AD, Pozzi N, Chen Z, Di Cera E (2014). Essential role of conformational selection in ligand binding. Biophys. Chem..

[29] Chakrabarti KS (2016). Conformational selection in a protein-protein interaction revealed by dynamic pathway analysis. Cell Rep..

[30] Reddy JG (2017). Simultaneous determination of fast and slow dynamics in molecules using extreme CPMG relaxation dispersion experiments. J. Biomol. NMR.

[31] Prinz J-H (2011). Markov models of molecular kinetics: generation and validation. J. Chem. Phys..

[32] Schütte C, Fischer A, Huisinga W, Deuflhard P (1999). A direct approach to conformational dynamics based on hybrid Monte Carlo. J. Comput. Phys..

[33] Pande VS, Beauchamp K, Bowman GR (2010). Everything you wanted to know about Markov state models but were afraid to ask. Methods.

[34] Buchete N-V, Hummer G (2008). Coarse master equations for peptide folding dynamics. J. Phys. Chem. B.

[35] Bowman GR, Beauchamp KA, Boxer G, Pande VS (2009). Progress and challenges in the automated construction of Markov state models for full protein systems. J. Chem. Phys..

[36] Olsson, S. Markov state models of protein-protein encounters. In *Protein Interaction. The Molecular Basis of Interactomics*. (eds Helms, V. & Kalinina, O.) (Wiley) in press.

[37] Olsson S, Wu H, Paul F, Clementi C, Noé F (2017). Combining experimental and simulation data of molecular processes via augmented Markov models. Proc. Natl. Acad. Sci. USA.

[38] Silva D-A, Bowman GR, Sosa-Peinado A, Huang X (2011). A role for both conformational selection and induced fit in ligand binding by the LAO protein. PLoS Comput. Biol..

[39] Gu S, Silva D-A, Meng L, Yue A, Huang X (2014). Quantitatively characterizing the ligand binding mechanisms of choline binding protein using markov state model analysis. PLoS Comput. Biol..

[40] Paul F, Noé F, Weikl TR (2018). Identifying conformational-selection and induced-fit aspects in the binding-induced folding of PMI from Markov state modeling of atomistic simulations. J. Phys. Chem. B.

[41] Plattner N, Doerr S, Fabritiis GD, Noé F (2017). Complete protein–protein association kinetics in atomic detail revealed by molecular dynamics simulations and Markov modelling. Nat. Chem..

[42] Philippe D (2011). Making ends meet: The importance of the N- and C-termini for the structure, stability, and function of the third SH3 domain of CIN85. Biochemistry.

[43] Smith CA (2016). Allosteric switch regulates protein-protein binding through collective motion. Proc. Natl. Acad. Sci. USA.

[44] Weikl TR, Paul F (2014). Conformational selection in protein binding and function. Protein Sci..

[45] Luz Z, Meiboom S (1963). Nuclear magnetic resonance study of the protolysis of trimethylammonium ion in aqueous solution—order of the reaction with respect to solvent. J. Chem. Phys..

[46] Bezsonova I (2008). Interactions between the three CIN85 SH3 domains and ubiquitin: implications for CIN85 ubiquitination. Biochemistry.

[47] He Y, Hicke L, Radhakrishnan I (2007). Structural basis for ubiquitin recognition by SH3 domains. J. Mol. Biol..

[48] E. W, Vanden-Eijnden E (2006). Towards a theory of transition paths. J. Stat. Phys..

[49] Metzner P, Schütte C, Vanden-Eijnden E (2009). Transition path theory for Markov jump processes. Multiscale Modeling & Simulation.

[50] Noé F, Schütte C, Vanden-Eijnden E, Reich L, Weikl TR (2009). Constructing the equilibrium ensemble of folding pathways from short off-equilibrium simulations. Proc. Natl. Acad. Sci. USA.

[51] Plattner N, Noe F (2015). Protein conformational plasticity and complex ligand-binding kinetics explored by atomistic simulations and Markov models. Nat. Commun..

[52] Thayer KM, Lakhani B, Beveridge DL (2017). Molecular dynamics-markov state model of protein ligand binding and allostery in CRIB-PDZ: Conformational selection and induced fit. J. Phys. Chem. B.

[53] Ge Y (2018). Simulations of the regulatory ACT domain of human phenylalanine hydroxylase (PAH) unveil its mechanism of phenylalanine binding. J. Biol. Chem..

[54] Paul F (2017). Protein-peptide association kinetics beyond the seconds timescale from atomistic simulations. Nat. Commun..

[55] Zhou G, Pantelopulos GA, Mukherjee S, Voelz VA (2017). Bridging microscopic and macroscopic mechanisms of p53-MDM2 binding with kinetic network models. Biophys. J..

[56] Collins AP, Anderson PC (2018). Complete coupled binding-folding pathway of the intrinsically disordered transcription factor protein Brinker revealed by molecular dynamics simulations and Markov state modeling. Biochemistry.

[57] Robustelli P, Piana S, Shaw DE (2020). Mechanism of coupled folding-upon-binding of an intrinsically disordered protein. J. Am. Chem. Soc..

[58] Michielssens S (2014). A designed conformational shift to control protein binding specificity. Angew. Chem. Int. Ed..

[59] Pérez-Hernández G, Paul F, Giorgino T, Fabritiis GD, Noé F (2013). Identification of slow molecular order parameters for Markov model construction. J. Chem. Phys..

[60] Schwantes CR, Pande VS (2013). Improvements in Markov state model construction reveal many non-native interactions in the folding of NTL9. J. Chem. Theory Comput..

[61] Best RB, Zheng W, Mittal J (2014). Balanced protein-water interactions improve properties of disordered proteins and non-specific protein association. J. Chem. Theory Comput..

[62] Rauscher S (2015). Structural ensembles of intrinsically disordered proteins depend strongly on force field: a comparison to experiment. J. Chem. Theory. Comput..

[63] Eaton WA (2000). Fast kinetics and mechanisms in protein folding. Annu. Rev. Biophys. Biomol. Struct..

[64] Snow CD, Nguyen N, Pande VS, Gruebele M (2002). Absolute comparison of simulated and experimental protein-folding dynamics. Nature.

[65] Carpino LA, Han GY (1972). 9-fluorenylmethoxycarbonyl amino-protecting group. J. Org. Chem..

[66] El Oualid F (2010). Chemical synthesis of ubiquitin, ubiquitin-based probes, and diubiquitin. Angew. Chem. Int. Ed..

[67] Wang S-S (1973). p-alkoxybenzyl alcohol resin andp-alkoxybenzyloxycarbonylhydrazide resin for solid phase synthesis of protected peptide fragments. J. Am. Chem. Soc..

[68] Knorr R, Trzeciak A, Bannwarth W, Gillessen D (1989). New coupling reagents in peptide chemistry. Tetrahedron Lett..

[69] Miranda LP, Alewood PF (1999). Accelerated chemical synthesis of peptides and small proteins. Proc. Natl. Acad. Sci. USA.

[70] Kabsch W (2010). XDS. Acta Crystallogr. D.

[71] McCoy AJ (2007). Phasercrystallographic software. J. Appl. Crystallogr..

[72] Vijay-kumar S, Bugg CE, Cook WJ (1987). Structure of ubiquitin refined at 1.8 Å resolution. J. Mol. Biol..

[73] Murshudov GN (2011). REFMAC5 for the refinement of macromolecular crystal structures. Acta Crystallogr. D.

[74] Emsley P, Lohkamp B, Scott WG, Cowtan K (2010). Features and development of Coot. Acta Crystallogr. D.

[75] Huang KY, Amodeo GA, Tong L, McDermott A (2011). The structure of human ubiquitin in 2-methyl-2, 4-pentanediol: A new conformational switch. Protein Sci..

[76] Ramage R (1994). Synthetic, structural and biological studies of the ubiquitin system: the total chemical synthesis of ubiquitin. Biochem. J..

[77] Johnson EC, Lazar GA, Desjarlais JR, Handel TM (1999). Solution structure and dynamics of a designed hydrophobic core variant of ubiquitin. Structure.

[78] Wolfram Research, Inc. Mathematica, Version 11.3 (Wolfram Research, Inc., Champaign, IL, 2018).

[79] Abraham MJ (2015). GROMACS: high performance molecular simulations through multi-level parallelism from laptops to supercomputers. SoftwareX.

[80] Lindorff-Larsen K (2010). Improved side-chain torsion potentials for the Amber ff99SB protein force field. Proteins Struct. Funct. Bioinf..

[81] Bussi G, Donadio D, Parrinello M (2007). Canonical sampling through velocity rescaling. J. Chem. Phys..

[82] Eastman P (2017). OpenMM 7: Rapid development of high performance algorithms for molecular dynamics. PLOS Comp. Biol..

[83] Wehmeyer, C. et al. Introduction to Markov state modeling with the PyEMMA software [article v1.0]. Living J. Comput. Mol. Sci. 1. 10.33011/livecoms.1.1.5965 (2019).

[84] Scherer MK (2015). PyEMMA 2: a software package for estimation, validation, and analysis of markov models. J. Chem. Theory Comput..

[85] McGibbon RT (2015). Mdtraj: a modern open library for the analysis of molecular dynamics trajectories. Biophysical Journal.

[86] Trendelkamp-Schroer B, Wu H, Paul F, Noé F (2015). Estimation and uncertainty of reversible Markov models. J. Chem. Phys..

[87] Röblitz S, Weber M (2013). Fuzzy spectral clustering by PCCA: application to Markov state models and data classification. Adv. Data. Anal. Classif..

[88] Chakrabarti, K. S. et al. High-power relaxation dispersion NMR data set at different ligand concentrations: a litmus test for classification of recognition mechanism. Edmond, V1. 10.17617/3.AVKYZC.

[89] Olsson, S. & Weikl, T. 1.68 milliseconds of MD simulation trajectories for the binding of ubiquitin to the SH3c domain from CIN85. Edmond, V1. 10.17617/3.8o.

