# Supplementary material for: A litmus test for classifying recognition mechanisms of transiently binding proteins
Source: Nat Commun. 2022 Jul 1;13:3792. doi: 10.1038/s41467-022-31374-5 (PMC9249894; doi:10.1038/s41467-022-31374-5)
Supplement: Supplementary file 1 — Supplementary Information [file 41467_2022_31374_MOESM1_ESM.pdf]

Supplementary Information

A litmus test for classifying recognition mechanisms of transiently binding proteins

Kalyan S. Chakrabarti,[1,2,*] Simon Olsson,[3,4,*] Supriya Pratihar,[2] Karin Giller,[2] Kerstin Overkamp,[2] Ko On Lee,[5] Vytautas Gapsys,[6] Kyoung-Seok Ryu,[5] Bert L. de Groot,[6] Frank Noé,[4,7,8] Stefan Becker,[2] Donghan Lee,[9,†] Thomas R. Weikl,[10,†] and Christian Griesinger[2,†]

[1] Division of Sciences, Krea University, Sri City, India
[2] Department of NMR Based Structural Biology, Max Planck Institute for Multidisciplinary Sciences, Göttingen, Germany
[3] Department of Computer Science and Engineering, Chalmers University of Technology, Gothenburg, Sweden
[4] Department of Mathematics and Computer Science, Freie Universität Berlin, Berlin, Germany
[5] Research Center for Bioconvergence Analysis, Korea Basic Science Institute, Ochang-Eup, Cheongju-Si, South Korea
[6] Department of Theoretical and Computational Biophysics, Max Planck Institute for Multidisciplinary Sciences, Göttingen, Germany
[7] Department of Physics, Freie Universität Berlin, Berlin, Germany
[8] Department of Chemistry, Rice University, Houston, TX, USA
[9] Department of Medicine, James Graham Brown Cancer Center, University of Louisville, Louisville, KY, USA
[10] Department of Biomolecular Systems, Max Planck Institute of Colloids and Interfaces, Potsdam, Germany

[*] These authors contributed equally to this work
[†] To whom correspondence should be addressed: donghan.lee@louisville.edu (D.L.), thomas.weikl@mpikg.mpg.de (T.R.W.), cigr@nmr.mpibpc.mpg.de (C.G.)

Contents

Supplementary Methods pages 2 to 12
Supplementary Figures 1 to 14 pages 13 to 26
Supplementary Tables 1 to 5 pages 27 to 32

Supplementary Methods

Determination of the binding affinity of wild-type and mutant ubiquitin with SH3c

The binding affinities of the wild-type and mutant ubiquitin proteins were measured using a series of ^{1}H, ^{15}N HSQC experiments in the 800 MHz Bruker spectrometers fitted with cryoprobe (TCI). A Total of 2048 and 256 complex points were collected in the ^{1}H and ^{15}N dimensions, respectively. Each increment was signal averaged by collecting 32 transients. In the titration experiments, the concentration of ^{15}N-labeled wild-type ubiquitin and G53A mutant (peptide-flip mode: out) were fixed at 400 μM. The concentration of the unlabeled SH3c was varied from 0, 40, 200, 400, 600, 800, 1200 to 1750 μM. In the case of the G53(D)Thr mutant (peptide-flip mode: in), the concentration of ^{15}N-labeled SH3c was kept constant at 400 μM and the (only E24 ^{15}N labeled, otherwise unlabeled) G53(D)Thr concentration was varied from 0, 40, 200, 400, 600, 800, 1200, 1750 to 2300 μM. In all cases, the buffer condition was the same as described in Methods. A set of 5 residues which were unaffected by binding were identified based on the criteria that they have the lowest standard deviations between chemical shift perturbation (CSP) values. The global uncertainty for the experimental data (to account for small changes in the solvent pH or ionic strength despite buffer matching) was determined as the average of the standard deviation of the set of 5 residues. The residue-specific uncertainty in CSP values were calculated by measuring the difference between the CSP values in free protein and at 40 μM ligand concentration (to account for pipetting and mixing of ligand errors at 10% of protein concentration). The largest of the (global or residue-specific) uncertainties are reported. The residue-wise data were globally fit to 1:1 binding model using the following equation

$$\Delta\delta_{\mathrm{obs}} = \Delta\delta_{\mathrm{max}}([\mathrm{P}]_0 + [\mathrm{L}]_0 + K_d) - \left[([\mathrm{P}]_0 + [\mathrm{L}]_0 + K_d)^2 - 4[\mathrm{P}]_0[\mathrm{L}]_0\right]^{1/2}/2[\mathrm{P}]_0 \qquad (1)$$

where $\Delta\delta_{\mathrm{obs}}$ is the observed chemical shift, $\Delta\delta_{\mathrm{max}}$ is the difference between the chemical shifts of the free and the bound states, $[\mathrm{P}]_0$ is the total protein concentration, $[\mathrm{L}]_0$ is the total ligand concentration, and K_d is the fitted dissociation constant.[1] The titration profile for the wild-type ubiquitin with SH3c is shown in Supplementary Fig. 6.

NMR data were processed using the NMRPipe/NMRDraw software suite.[2] Peak picking and peak intensities were extracted using NMRPipe. The spectra were visualized in NMRpipe, Topspin (Bruker Biospin corporation), and Sparky (Goddard and Kneller, 2008, SPARKY3, UCSF). Protein structures were visualized using Chimera[3] and PyMOL (Schrödinger, LLC.).

Exchange rates k_{ex} for two-state binding, induced fit, and conformational selection in high-power relaxation dispersion NMR experiments

Our high-power relaxation dispersion NMR experiments provide kinetic information on protein binding in equilibrium. The concentrations of the unbound and bound protein P and ligand L are therefore constant and equal to their equilibrium concentrations. A consequence is that the reaction schemes of protein binding in the relaxation dispersion NMR experiments are of first order

for all total concentrations $[\mathrm{P}]_0$ and $[\mathrm{L}]_0$ of the protein P and ligand L. In this regard, the NMR experiments are different from chemical relaxation experiments such as stopped-flow mixing or temperature-jump experiments in which the concentrations of the unbound and bound protein species are time-dependent. The reaction schemes of chemical relaxation experiments are of second order and can only be approximated as 'pseudo-first order' if the total concentration of one of the binding partners greatly exceeds the concentration of the other partner.[4]

For a **two-state binding** process

$$\mathrm{P} + \mathrm{L} \underset{k_{\mathrm{off}}}{\overset{k_{\mathrm{on}}}{\rightleftharpoons}} \mathrm{PL} \tag{2}$$

the two-state exchange rate k_{ex} for atoms of the protein P obtained from relaxation dispersion experiments corresponds to the relaxation rate of the binding process that is probed in the experiments:

$$k_{\mathrm{ex}} = k_{\mathrm{on}}[\mathrm{L}]_{\mathrm{eq}} + k_{\mathrm{off}} \tag{3}$$

The exchange rate k_{ex} for atoms of the protein P depends on the equilibrium concentration $[\mathrm{L}]_{\mathrm{eq}}$ of the unbound ligand L, because the rate $k_{\mathrm{on}}[\mathrm{L}]_{\mathrm{eq}}$ from the unbound to the bound state of a protein molecule is proportional to $[\mathrm{L}]_{\mathrm{eq}}$. The concentration $[\mathrm{L}]_{\mathrm{eq}}$ in turn is determined by the dissociation constant K_d and the total concentrations $[\mathrm{P}]_0$ and $[\mathrm{L}]_0$ of the protein P and ligand L:

$$[\mathrm{L}]_{\mathrm{eq}} = \frac{1}{2}\Big([\mathrm{L}]_0 - [\mathrm{P}]_0 - K_d + \sqrt{([\mathrm{L}]_0 - [\mathrm{P}]_0 + K_d)^2 + 4[\mathrm{P}]_0 K_d}\Big) \tag{4}$$

This equation follows from the definition $K_d = [\mathrm{P}][\mathrm{L}]/[\mathrm{PL}]$ of the dissociation constant and the mass balance equations $[\mathrm{P}] + [\mathrm{PL}] = [\mathrm{P}]_0$ and $[\mathrm{L}] + [\mathrm{PL}] = [\mathrm{L}]_0$. For the two-state binding process, the exchange rate k_{ex} increases with increasing total ligand concentration $[\mathrm{L}]_0$, because the equilibrium concentration $[\mathrm{L}]_{\mathrm{eq}}$ of the unbound ligand increases with $[\mathrm{L}]_0$.

For a **conformational-selection** process

$$\mathrm{P}_1 + L \underset{k_{21}}{\overset{k_{12}}{\rightleftharpoons}} \mathrm{P}_2 + L \underset{k_-}{\overset{k_+}{\rightleftharpoons}} \mathrm{P}_2\mathrm{L} \tag{5}$$

the two-state exchange rate k_{ex} for atoms of the protein P corresponds to the dominant relaxation rate of the first-order process

$$\begin{aligned} k_{\mathrm{ex}} =& \frac{1}{2}\Big(k_{12} + k_{21} + k_+[\mathrm{L}]_{\mathrm{eq}} + k_- \\ & - \sqrt{(k_{12} + k_{21} + k_+[\mathrm{L}]_{\mathrm{eq}} + k_-)^2 - 4(k_{12}(k_+[\mathrm{L}]_{\mathrm{eq}} + k_-) + k_- k_{21})}\Big) \end{aligned} \tag{6}$$

with $[\mathrm{L}]_{\mathrm{eq}}$ as in Eq. (4) and the overall dissociation constant

$$K_d = \frac{k_-(k_{12} + k_{21})}{k_+ k_{12}} \tag{7}$$

of conformational-selection binding.[4] The exchange rate k_{ex} of the conformational-selection process increases with increasing total ligand concentration $[\text{L}]_0$ if the conformational exchange rate k_{12} is larger than the unbinding rate k_-, and decreases with $[\text{L}]_0$ for $k_{12} < k_-$.

For an **induced-fit** process

$$\text{P}_1 + L \underset{k_-}{\overset{k_+}{\rightleftharpoons}} \text{P}_1\text{L} \underset{k_{21}}{\overset{k_{12}}{\rightleftharpoons}} \text{P}_2\text{L} \tag{8}$$

the two-state exchange rate k_{ex} corresponds to the dominant relaxation rate

$$\begin{aligned} k_{\text{ex}} =& \frac{1}{2}\Big(k_+[\text{L}]_{\text{eq}} + k_- + k_{12} + k_{21} \\ &- \sqrt{(k_+[\text{L}]_{\text{eq}} + k_- + k_{12} + k_{21})^2 - 4(k_+[\text{L}]_{\text{eq}}(k_{12} + k_{21}) + k_{21}k_-)}\Big) \end{aligned} \tag{9}$$

of the first-order process with $[\text{L}]_{\text{eq}}$ as in Eq. (4) and the overall dissociation constant

$$K_d = \frac{k_- k_{21}}{k_+(k_{21} + k_{12})} \tag{10}$$

of induced-fit binding.[4] The exchange rate k_{ex} of the induced-fit process increases with increasing total ligand concentration $[\text{L}]_0$.

Fitting of k_{ex} data for ubiquitin

We focus on ubiquitin residue positions for which the exchange rate k_{ex} in the presence of SH3c is clearly smaller than the exchange $k_{\text{ex}}(0) \simeq 20\,000\,\text{s}^{-1}$ in the absence of SH3c.[5] The exchange rate at these residues positions reflects the binding reaction. To analyse this binding reaction, we selected those residues positions for which the k_{ex} value including error is smaller than $10\,000\,\text{s}^{-1}$ at least at four out of the six SH3c concentrations $0.02, 0.05, 0.1, 0.25, 0.5$, and $1\,\text{mM}$ at which the relaxation dispersion experiments were conducted. These residue positions are the 22 residues 5, 6, 7, 8, 10, 13, 14, 41, 42, 43, 44, 45, 46, 48, 49, 50, 51, 66, 67, 69, 71, and 72 out of the total 55 residue positions (Supplementary Table 2 and Supplementary Figs. 2, 3, and 7 to 12).

We fitted the data for k_{ex} as a function of the SH3c concentration at the selected 22 residues positions with the functions (3), (6), and (9) of the two-state binding, conformational-selection, and induced-fit models. The k_{ex} values as a function of the SH3c concentration were fitted individually for each of the 22 residue positions. All fits were performed with the function NonlinearModelFit of Mathematica 11.3 for the ubiquitin concentration $1\,\text{mM}$ of the experiments. In these fits, we made use of the experimentally determined value $K_d = 370 \pm 5\,\mu\text{M}$ for the dissociation constant. As usual, the data points for k_{ex} were weighted by the inverse square of their errors in all fits, which restricts the impact of outliers with large errors. Our fits of the two-state binding function (3) with Eq. (4) are one-parameter fits with fit parameter k_{off} after replacing k_{on} by k_{off}/K_d in Eq. (3).

The function $k_{\text{ex}}([\text{SH3}])$ of the conformational-selection model has three parameters after replacing k_{on} based on Eq. (7) and the experimentally known value of K_d (see Eq. 6). These parameters are the conformational exchange rates k_{12} and k_{21} and the unbinding rate k_-. A constraint in model fitting is that the sum $k_{12} + k_{21}$ of the conformational exchange rates needs to be larger or equal to exchange rate $k_{\text{ex}}(0) \simeq 20\,000\,\text{s}^{-1}$ obtained from experiments in the absence of SH3c.[5] Three-parameter fits of k_{12}, k_{21}, and k_- to the experimentally determined k_{ex} values at the different residue positions and SH3c concentrations do not lead to definite values of k_{21}. To obtain reliable fit values of k_{12} and k_-, we determined the values of these parameters in two-parameter fits for different, constrained values of $k_{12}+k_{21}$ between $20\,000\,\text{s}^{-1}$ and $200\,000\,\text{s}^{-1}$. We find that the fit results for k_{12} and k_- are practically independent of the chosen, constrained value for $k_{12}+k_{21}$, i.e. the differences in the fit results for difference choices of $k_{12} + k_{21}$ are much smaller than the fit errors. We determined the fit errors of k_{12} and k_- as the maximum of two independent error estimates, (1) from the fitting weights $(1/\Delta k_{ex})^2$ with a variance estimator function of 1 in Mathematica, and (2) as standard jackknife (JK) error $\sqrt{(n-1/n)\sum_{i=1}^{n}(k_i^{\text{JK}}-k)^2}$ where n is the number of data points, k_i^{JK} is the fit result without data point i, and k is the fit result with all data points.

In Supplementary Fig. 3, the resulting fits of the two-state binding and conformational-selection model at the 22 selected residues positions are shown as blue and red lines, respectively. The shaded blue region is the area enclosed by the n jackknife fits of the two-state binding model for all subsets with $n-1$ data points, and the shaded red region is the area enclosed by the corresponding jackknife fits of the conformational-selection model. The relative quality of the one-parameter fits of the two-state binding model and the two-parameter fits of the conformational-selection model can be assessed with the finite-size corrected Akaike information criterion (AICc).[6] The AICc is founded in information theory and provides an estimate of the relative quality of fit models with different number of parameters, in a tradeoff between goodness of fit and simplicity of the models. In general, the relative likelihood of two models with AICc values A_1 and A_2 is $\exp[(\min[A_1, A_2] - A_i)/2]$ with $i = 1$ or 2. For assessing the relative quality of the fits in Supplementary Fig. 3, it is important to note that the conformational-selection model includes the two-state binding model as a special case. The goodness of fit of the conformational-selection model in terms of fit residuals is therefore better or equal to the goodness of fit of the two-state binding model. Smaller AICc values for fits of the two-state binding model indicate that these fits are "more economic" because they require only one fit parameter, compared to the two fit parameters of the conformational-selection model. In contrast, smaller AICc values for fits of the conformational-selection model indicate that the additional fit parameter of this model is required to achieve a good fit with small fit residuals. The AICc values for the fits of the two-state binding and conformational-selection model are specified as blue and red numbers in the top left corner of the subfigures of Supplementary Fig. 3. In addition to the AICc values of the individual fits, we have determined overall AICc values for the two-state binding and conformational-selection model by combining the data and fit parameters of these 22 fits into one overall fit in the program Mathematica 11.3. The parameter values and errors obtained from this overall fit are identical to the values and errors of the individual fits shown in Supplementary Fig. 3. The resulting overall AICc values are 2435.9 for the conformational-selection model and 2785.7 for the two-state binding model, which leads

to a relative likelihood of practically 0 for the two-state binding model. Our systematic model selection using the Aikake information criterion thus rules at the two-state binding model for ubiquitin, which can also be confirmed by multiplying the relative likelihoods of the models obtained from the individual AICc values of the 22 fits. In contrast to the two-state binding model, the conformational-selection model provides good fits to the data points at all residue positions of Supplementary Fig. 3. The values for the fit parameters k_{12} and k_- of the conformational-selection model at the different residue positions are overall consistent with each other within the fitting errors (Fig. 2e,f), which indicates a consistent, global conformational-selection binding mechanism for ubiquitin.

The dashed red lines in Supplementary Fig. 3 represent fit results for the induced-fit model. The fits are two-parameter fits of the model parameters k_{21} and k_- for constrained values of $k_{12} + k_{21}$ between $20\,000\,\mathrm{s}^{-1}$ and $200\,000\,\mathrm{s}^{-1}$ as in the case of the conformational-selection model. The parameter k_{on} has again been replaced based on Eq. (10) and the experimentally known value of K_d (see Eq. 6). The error estimates of the two fit parameters greatly exceed the parameter values, which indicates that the two-parameter fit of the induced-fit model is underdetermined. The fitted functions $k_{\mathrm{ex}}([\mathrm{SH3}])$ for the induced-fit model are essentially overlapping with the functions from one-parameter fits of the two-state binding model, which is included in the induced-fit model as a special case. The fits of the induced-fit model thus "reduce" to the two-state binding model. Because of the parameter uncertainties, we used the global search method "DifferentialEvolution" in fitting the induced-fit model with Mathematica.

Fitting of k_{ex} data for SH3c

For SH3c, the exchange rate k_{ex} is clearly affected by the presence of ubiquitin at the 12 residue positions 269, 275, 276, 278, 280, 282, 283, 303, 305, 306, 322, and 324 (Supplementary Table 3 and Supplementary Figs. 2 and 4). At these residue positions, the k_{ex} value including error is smaller than $10\,000\,\mathrm{s}^{-1}$ at least at four out of the eight ubiquitin concentrations $0.02, 0.05, 0.05, 0.1, 0.15, 0.25, 0.5$, and $1\,\mathrm{mM}$ at which the relaxation dispersion experiments were conducted (Supplementary Table 3).

The SH3c k_{ex} data can be well fitted by the two-state binding model with the single fit parameter k_{off} after replacing k_{on} by k_{off}/K_d in Eq. (3). The fits and shaded red error regions shown in Supplementary Fig. 4 were obtained in the same way as for the ubiquitin data (see previous section). Two-parameter fits of the SH3c k_{ex} data with the conformational-selection and induced-fit models are underdetermined, i.e. the error estimates of the two fit parameters greatly exceed the parameter values. Because the conformational-selection and induced-fit models include the two-state binding model as special case, the residuals for two-parameter fits of these models do not exceed the residuals of one-parameter fits of the two-state binding model. Therefore, the k_{ex} data for SH3c are consistent with the two-state model, but do not exclude the conformational-selection and induced-fit models. An increase of k_{ex} with increasing ligand concentration is compatible with all three models (Fig. 1), in contrast to the decrease of k_{ex} with increasing ligand concentration observed for the ubiquitin k_{ex} data.

Markov modeling details

Filtering of discrete MD trajectories: We have a set of discrete state trajectories, $\mathcal{T} = \{T_0, \ldots, T_K\}$, where each trajectory, $T_i = \{s_0, s_1, \ldots, s_{N_i}\}$ has N_i time-steps. At each time-step, i, the trajectory is in one of M disjoint Markov states $s_i \in \{\mathcal{S}_1, \ldots, \mathcal{S}_M\}$. We assume uniformly sampled time-points spaced by the time Δt. We define a filter $\mathcal{F}(T_i; \tau)$ which maps each trajectory to a filtered version $T_i^{\mathcal{F}}$ where each step now adopts a subset of the M Markov states. In the current work, we define the 'most-frequent' filter:

$$\mathcal{F}(T;\tau) = \left\{ \arg\max \left(\sum_{W_i} \delta(\mathcal{S}_1 - W_{i,j}), \ldots, \sum_{W_i} \delta(\mathcal{S}_M - W_{i,j}) \right) \right\}_{W_{i=0}^{N_i}} \quad (11)$$

where W_i is a window at the $i\,\mathrm{th}$ time-point, and δ is Diracs' delta function. The window W_i at time-point i is defined as all time-points, j, in the range ($i - \tau/2$;$i + \tau/2$), where τ is the lag-time in units of Δt. If $i > \tau/2$ or $i < \tau/2$ the window is truncated at the trajectory limits. The 'most-frequent' filter assigns the state of a trajectory at time, i, to the state which was most frequently visited in the time-window, ($i - \tau/2$;$i + \tau/2$). The filtered trajectories helps us prune short-lived states and compensate for barrier recrossing events due to discretization and projection errors.

Analysis of Markov state model: The fully bound state is the metastable state with highest probability (state 14, 39%), which coincides with the state with lowest average RMSD to a model built using PRE and RDC restraints $\overline{\mathrm{RMSD}}_{\mathrm{2K6D}} = 0.38^{0.45}_{0.33}$ nm[7] and an experimental model of the ubiquitin:Sla-SH3c complex $\overline{\mathrm{RMSD}}_{\mathrm{2JT4}} = 0.21^{0.31}_{0.15}$ nm.[8] Sla1 SH3c is a low sequence identity homolog to the SH3c domain studied here. The two experimental structures differ slightly in their orientation of the ubiquitin and SH3c domains. The bound state in the MSM encompasses both conformations. However, the conformation consistent with the Sla1 SH3c domain appears slightly more stable in the force field used in this study. To test whether the ubiquitin:Sla-SH3c state was more stable, we carried out further simulations started from the experimental coordinates of 2K6D using the same simulation setup. The system relaxes to an orientation consistent with the Sla1 SH3c complex structure within tens of nanoseconds. We used a transition path theory (TPT)[9,10] based analysis to investigate the binding mechanism from unbound metastable states to the fully bound state discussed above. TPT gave us access to the binding-committor probabilities p_{bind} and flux along association pathways shown in Fig. 3.

Assignment of Markov states to bound and unbound states: To compute the macroscopic dissociation constant, K_d, and on- and off-rates, we need a definition of "unbound" and "bound" states. To gauge the robustness of the K_d to this definition, we vary it using a threshold on the committor probability p_{bind} as defined above. If p_{bind} is less than the threshold it is included in the unbound state and vice versa for the bound state. We vary the threshold in the open interval 0 to 1 and compute macroscopic K_d, and on- and off-rates as a function of the threshold. A threshold of $p_{\mathrm{bind}} = 0.57$, which is located within a plausible transition state region, yields a K_d value that matches the experimental value. However,the entire range of predicted K_d values for different threshold choices is within the expected error of current state-of-the-art force fields

(Fig. 6).

Peptide-flip populations: We compute the peptide-flip mode via the procedure previously described.[5] In the fully bound state with $p_{\text{bind}} = 1$, the peptide-flip populations found in the MD simulations are:

$p_{\text{in}} = 0.02_{0.01}^{0.03}$ and $p_{\text{out}} = 0.98_{0.97}^{0.99}$

In the intermediate state with $0.45 < p_{\text{bind}} < 0.75$, the peptide-flip populations are:

$p_{\text{in}} = 0.02_{0.01}^{0.04}$ and $p_{\text{out}} = 0.98_{0.96}^{0.99}$.

The corresponding values for the unbound state, estimated using a separate set of simulations, are reported in the following section.

Pincer mode populations: We compute the pincer mode using the procedure previously described:[11] In the fully bound state with $p_{\text{bind}} = 1$, the pincer mode populations found in the MD simulations are:

$p_{\text{open}} = 0.33_{0.29}^{0.38}$ and $p_{\text{closed}} = 0.66_{0.62}^{0.71}$

In the intermediate $0.45 < p_{\text{bind}} < 0.75$, the pincer mode populations are:

$p_{\text{open}} = 0.62_{0.58}^{0.65}$ and $p_{\text{closed}} = 0.38_{0.35}^{0.42}$

C-terminal mode populations: In the fully bound state with $p_{\text{bind}} = 1$, the C-terminal populations found in the MD simulations are:

$p_{\text{compact}} = 0.058_{0.043}^{0.078}$ and $p_{\text{extended}} = 0.942_{0.922}^{0.958}$

In the intermediate $0.45 < p_{\text{bind}} < 0.75$, the C-terminal populations are:

$p_{\text{compact}} = 0.024_{0.017}^{0.034}$ and $p_{\text{extended}} = 0.976_{0.966}^{0.983}$

Molecular dynamics simulations and modeling of unbound ubiquitin

In the simulations of ubiquitin and SH3c binding, the unbound state is only marginally stable. Consequently, to robustly quantify thermodynamic and kinetic properties of conformational modes in ubiquitin in the unbound state, we carried out simulations in the absence of SH3c, to represent a fully unbound state. The results presented in this section are based on a total of 101.4 μs of ubiquitin in water, from 18 independent simulations. Simulations were seeded from representative configurations in the binding simulation (see below).

Peptide flip: We built a two-state Bayesian hidden Markov model (HMM) (1000 posterior samples), with a lag time of $8.2\,\text{ns}$, with two state clustering using only the peptide-flip mode (definition is given in[5]) as feature, for consistency with previous results. We use this model to compute the following physical properties to characterize the comformational mode:

$\Delta G_{\text{in}\to\text{out}} = -9.3_{-10.6}^{-7.98}\,\text{kJ} \cdot \text{mol}^{-1}$

$k_{\text{in}\to\text{out}} = 11_{8}^{16} \cdot 10^{6}\, s^{-1}$ and $k_{\text{out}\to\text{in}} = 27_{17}^{39} \cdot 10^{4}\, s^{-1}$

$p_{\text{in}} = 0.02_{0.01}^{0.04}$ and $p_{\text{out}} = 0.98_{0.96}^{0.99}$

All estimates are robust. In agreement with previous simulation results,[5] the "out" conformation of the peptide-flip mode clearly dominates over the "in" conformation in our model.

The populations in the "in" and "out" conformations determined from simulations thus deviate from the experimental result that both peptide-flip conformations are equally populated (Fig. 5h,i). In general, the populations of alternative conformations are difficult to estimate from simulations, because systematic errors of few $\mathrm{kJ} \cdot \mathrm{mol}^{-1}$ in state-of-the-art molecular dynamics force-fields[12,13] can lead to large deviations in populations.

Pincer mode: We built a two-state Bayesian hidden Markov model (HMM) (1000 posterior samples), with a lag time of $8.2\,\mathrm{ns}$, with two state clustering using only the pincer mode (definition is given in[11]) as feature, for consistency with previous results. We use this model to compute the following physical properties to characterize the comformational mode:

$\Delta G_{\text{open}\to\text{closed}} = 0.1_{-0.1}^{0.2}$ kJ · mol^{-1}

$k_{\text{open}\to\text{closed}} = 15_{7}^{24} \cdot 10^{6}\, s^{-1}$ and $k_{\text{closed}\to\text{open}} = 16_{11}^{24} \cdot 10^{6}\, s^{-1}$

$p_{\text{open}} = 0.51_{0.49}^{0.52}$ and $p_{\text{closed}} = 0.49_{0.47}^{0.51}$

All estimates are robust.

C-terminal mode: C-term mode is defined by a TICA transformation of the sines and cosines of back-bone torsion angles of the Ubiquitin C-terminus (details above). The C-term mode is broadly characterized by salt-bridge formation and breaking between the C-term carbonic acid and Arg72 (Fig. 3). This movement is characterized by compact (formed/low) and extended (broken/high) states (cut-off in the first TIC is at -0.5). The extended state is more flexible compared to the compact state.

We estimate a two-state Bayesian HMM (1000 posterior samples), with lag-time of $18.6\,\mathrm{ns}$, with twelve state clustering using the two dominant TICs of the cosines and sines of all back-bone torsions of residues 69-76. We use this model to compute physical properties of the C-term mode:

$\Delta G_{\text{extended}\to\text{compact}} = 3.4_{2.8}^{4}$ kJ · mol^{-1}

$k_{\text{extended}\to\text{compact}} = 1.7_{1.4}^{2.1} \cdot 10^{6}\, s^{-1}$ and $k_{\text{compact}\to\text{extended}} = 6.6_{5.4}^{7.9} \cdot 10^{6}\, s^{-1}$

$p_{\text{extended}} = 0.79_{0.76}^{0.83}$ and $p_{\text{compact}} = 0.21_{0.17}^{0.24}$

Comparison of simulation and modeling results to experimental data

Paramagnetic relaxation enhancements: We compare to previously reported paramagnetic relaxation enhancement (PRE) derived distances[7] as a secondary means of model validation. We use the following weighted average:

$$\langle d \rangle_{\text{MSM}} = \left(\sum_{M} \pi_M d_M^{-6} \right)^{-\frac{1}{6}} \tag{12}$$

where π_M is the state probability of Markov state M, and $d_M = \left(\frac{1}{\mathcal{S}_M} \sum_{m \in \mathcal{S}_M} d_m^{-6} \right)^{-\frac{1}{6}}$, where d_m is the distance between the spin label and a measured resonance, and $\mathcal{S}_M$ denotes all frames assigned to Markov state M. The reported PRE-derived distances are calibrated to be sensitive

to only the bound state population. Therefore, we evaluated the root mean square error between the computed PRE distances from the MSM and the experimentally reported values, varying the population of the unbound state ($p_{\mathrm{bind}} = 0$) between 0 to 100 %, where 100 % corresponds to fully unbound and 0 % corresponds to only including states with $p_{\mathrm{bind}} > 0$. We perform this perturbation by rescaling MSM stationary probabilities of the unbound state by a positive scalar and renormalizing the stationary distribution. We find 0 % unbound state gives the best agreement with experiment (Supplementary Fig. 5)

Order parameters. To evaluate the changes in fast time-scale flexibility along the ubiquitin:SH3c binding process, we computed the S^2 order parameters, reporting on local angular flexibility of the backbone amide, $\mathrm{H-N}$. We speculate, since the extended C-terminus (and more flexible) state is higher populated in the bound state, it will manifest as lower order parameters in the bound compared to the unbound state. The $\mathrm{H-N}$ order parameter S^2 is back-computed from the Markov model using the infinite time limit of the angular correlation function $C_I(\tau)$ which can be computed using[14]

$$S^2 = \frac{3}{2}\left(\langle x^2\rangle^2 + \langle y^2\rangle^2 + \langle z^2\rangle^2 + \langle xy\rangle^2 + \langle xz\rangle^2 + \langle yz\rangle^2\right) - \frac{1}{2} \tag{13}$$

where x, y, and z are the elements of the unit vector of the $\mathrm{H-N}$ bond and angular brackets denote ensemble averages weighted according to the Markov model consistent with the selected committor probability p_{bind}, e.g. bound, unbound, and intermediate states. The unbound and bound states here are fully unbound and bound states in the model, with $p_{\mathrm{bind}} = 0$ and $p_{\mathrm{bind}} = 1$, respectively, and the intermediate states are states with $0.45 < p_{\mathrm{bind}} < 0.75$. We find that the C-terminus of the bound state is indeed more flexible (Supplementary Fig. 14). We compare to experimental transverse relaxation rates R_2, their ratio in between the unbound and bound states, as well as an approximate order parameter, computed by normalizing the R_2 by highest rate (Supplementary Fig. 14).

Population shifts of ubiquitin peptide flip mode during binding

The population shift of the peptide flip mode of ubiquitin during binding can be estimated from the dissociation constants of the mutants G53A and G53(D)T that lock ubiquitin almost fully into the "out" and "in" conformation of the peptide flip, respectively. In a two-state model of the peptide flip mode with "in" conformation $\mathrm{P_{in}}$ and "out" conformation $\mathrm{P_{out}}$, the overall concentration of unbound ubiquitin can be expressed as $[\mathrm{P_{in}}] + [\mathrm{P_{out}}]$, and the overall concentration of the bound protein as $[\mathrm{P_{in}L}] + [\mathrm{P_{out}L}]$. Here, L stands for the CIN85 SH3c domain as ligand. The overall dissociation constant then is:

$$K_d = \frac{([\mathrm{P_{in}}] + [\mathrm{P_{out}}])\,[\mathrm{L}]}{[\mathrm{P_{in}L}] + [\mathrm{P_{out}L}]} \tag{14}$$

Our aim is to determine the two dissociation constants for the out and in conformation, defined as:

$$K_{\mathrm{in}} = \frac{[\mathrm{P_{in}}][\mathrm{L}]}{[\mathrm{P_{in}L}]} \tag{15}$$

$$K_{\text{out}} = \frac{[\text{P}_{\text{out}}][\text{L}]}{[\text{P}_{\text{out}}\text{L}]} \tag{16}$$

The last two equations lead to

$$\frac{x_u}{x_b} = \frac{K_{\text{in}}}{K_{\text{out}}} \tag{17}$$

where $x_u = [\text{P}_{\text{in}}]/[\text{P}_{\text{out}}]$ and $x_b = [\text{P}_{\text{in}}\text{L}]/[\text{P}_{\text{out}}\text{L}]$ are the relative populations of the out and in conformation in the unbound and bound state of ubiquitin. Eq. (17) indicates that the ratio of x_u and x_b, which is a measure for the population shift during binding, is equal to the ratio of the dissociation constants K_{out} and K_{in} of the two conformations. Because the mutants G53(D)T and G53A lock ubiquitin almost fully into the "in" and "out" conformation, the dissociation constants of the "in" and "out" conformation can be estimated as $K_{\text{in}} \simeq K_d^{\text{G53(D)T}} = 374 \pm 48\,\mu\text{M}$ and $K_{\text{out}} \simeq K_d^{\text{G53A}} = 537 \pm 28\ \mu\text{M}$, which leads to the population shift $x_u/x_b = K_{\text{in}}/K_{\text{out}} = 0.65 \pm 0.09$.

In the Markov state model, the population shift of the peptide-flip mode can be estimated as $x_u/x_b = 0.91(\text{CI95\%} : 0.461 - 1.775)$, which agrees with the population shift value obtained from the mutational data within the numerical accuracy.

References

[1] Williamson, M. P. Using chemical shift perturbation to characterise ligand binding. *Prog. Nucl. Magn. Reson. Spectrosc.* **73**, 1–16 (2013).

[2] Delaglio, F. *et al.* NMRPipe: A multidimensional spectral processing system based on UNIX pipes. *J. Biomol. NMR* **6** (1995).

[3] Pettersen, E. F. *et al.* UCSF chimera – a visualization system for exploratory research and analysis. *J. Comput. Chem.* **25**, 1605–1612 (2004).

[4] Paul, F. & Weikl, T. R. How to distinguish conformational selection and induced fit based on chemical relaxation rates. *PLoS Comput. Biol.* **12**, e1005067 (2016).

[5] Smith, C. A. *et al.* Allosteric switch regulates protein-protein binding through collective motion. *Proc. Natl. Acad. Sci. USA* **113**, 3269–3274 (2016).

[6] Cavanaugh, J. E. Unifying the derivations of the Akaike and corrected Akaike information criteria. *Stat. Probabil. Lett.* **31**, 201–208 (1997).

[7] Bezsonova, I. *et al.* Interactions between the three CIN85 SH3 domains and ubiquitin: Implications for CIN85 ubiquitination. *Biochemistry* **47**, 8937–8949 (2008).

[8] He, Y., Hicke, L. & Radhakrishnan, I. Structural basis for ubiquitin recognition by SH3 domains. *J. Mol. Biol.* **373**, 190–196 (2007).

[9] E., W. & Vanden-Eijnden, E. Towards a theory of transition paths. *J. Stat. Phys.* **123**, 503–523 (2006).

[10] Metzner, P., Schütte, C. & Vanden-Eijnden, E. Transition path theory for Markov jump processes. *Multiscale Modeling & Simulation* **7**, 1192–1219 (2009).

[11] Lange, O. F. *et al.* Recognition dynamics up to microseconds revealed from an RDC-derived ubiquitin ensemble in solution. *Science* **320**, 1471–1475 (2008).

[12] Best, R. B., Zheng, W. & Mittal, J. Balanced protein-water interactions improve properties of disordered proteins and non-specific protein association. *J. Chem. Theory Comput.* **10**, 5113–5124 (2014).

[13] Rauscher, S. *et al.* Structural ensembles of intrinsically disordered proteins depend strongly on force field: A comparison to experiment. *J. Chem. Theory. Comput.* **11**, 5513–24 (2015).

[14] Chatfield, D. C., Szabo, A. & Brooks, B. R. Molecular dynamics of staphylococcal nuclease: Comparison of simulation with 15N and 13C NMR relaxation data. *J. Am. Chem. Soc.* **120**, 5301–5311 (1998).

Supplementary Figures

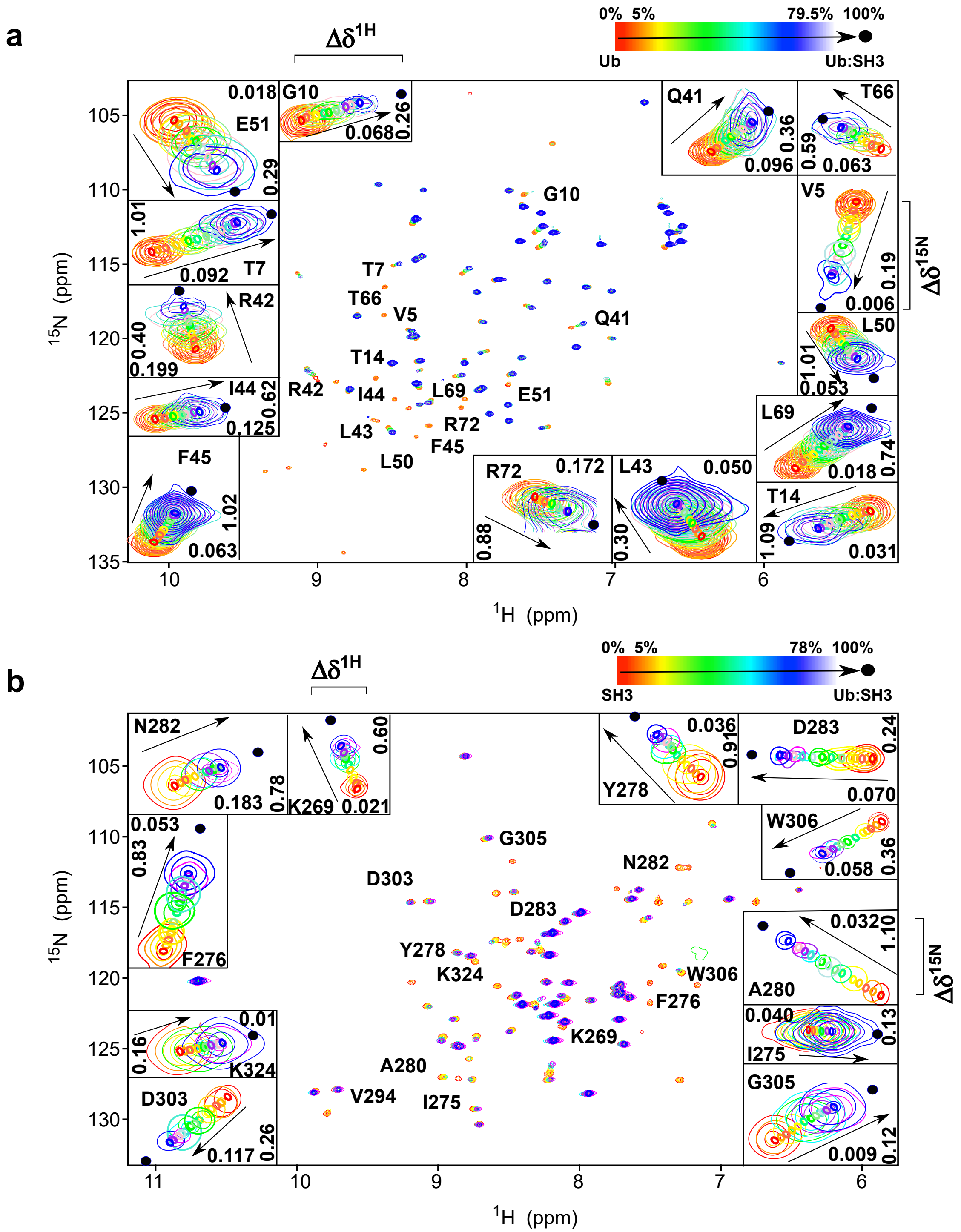

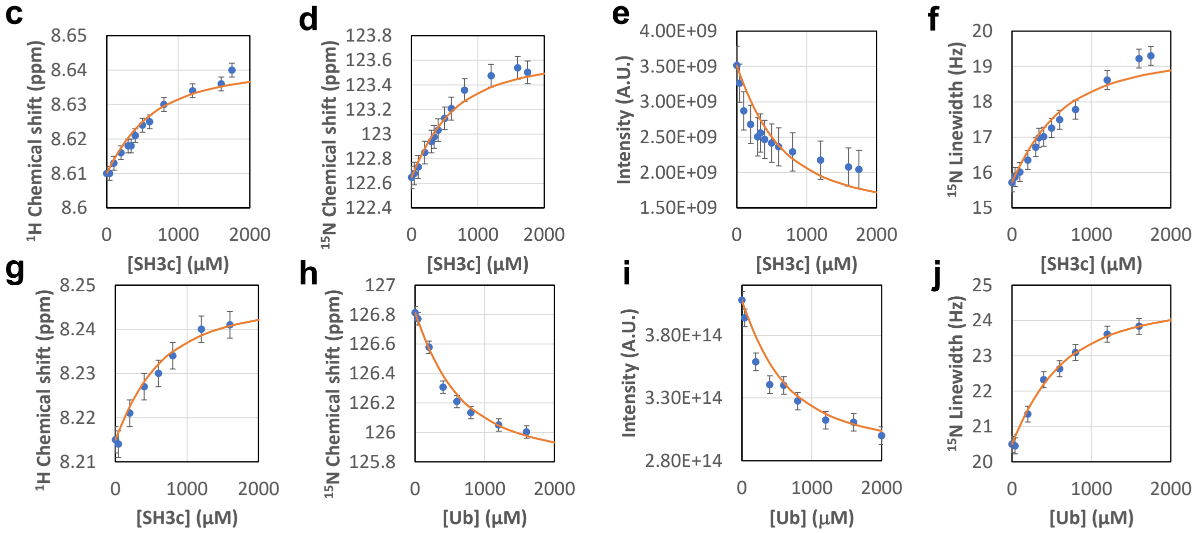

Supplementary Figure 1: **Chemical shift changes on ubiquitin and SH3c upon binding.** **a** ^{1}H –^{15}N HSQC titration of ^{15}N ubiquitin with unlabeled SH3c for the ratio of the complex ranging from 0 (red) to 79.5% (blue). The color of the cross peaks changes gradually from red (free) to blue (bound) as the concentration of the complex increases. The innermost contour is drawn with a thicker stroke to highlight the peak position. Exchange between free ubiquitin and the complex is fast (black arrows are shown in the inset as visual guidance) on the chemical shift timescale. The concentration of the complex was determined from the dissociation constant $K_D = 370 \pm 15$ μM, which was calculated from fitting the chemical shifts observed for ubiquitin resonances over the whole range of SH3c concentrations (see Supplementary Fig. 6). The subset of residues, which were analyzed for binding dynamics, are marked on the spectra and zoomed in the inset figures. The $\Delta\delta^{1\mathrm{H}}$ (x-axis) and $\Delta\delta^{15\mathrm{N}}$ (y-axis) values - in the insets - were calculated from the simultaneous fitting of the ^{1}H and ^{15}N chemical shifts. The position of the 100% complex is represented as the back circles in the spectra with radii corresponding to errorbars. **b** The reverse titration was performed on ^{15}N-labeled SH3c by titrating in unlabeled ubiquitin such that the complex ranged from 0 (red) to 78% (blue). Similar to **a**, the exchange between free SH3c and the complex is fast on the chemical shift timescale. The residues experiencing the largest differences in chemical shift along the ^{15}N dimension, T14 of ubiquitin (**c-f**) and A280 of SH3c (**g-j**) show gradual change in peak positions along ^{1}H dimensions (**c, g**) and ^{15}N dimensions (**d, h**) in ppm, peak intensities in arbitrary units (a.u.) (**e, i**) and ^{15}N linewidths in Hz (**f, j**). The titration curve (orange) corresponds to the global K_D of 370 μM. Global ($n = 5$) and residue-specific uncertainties in the datapoints were estimated as described in Supplementary Methods. The largest of the (global or residue-specific) uncertainties are reported. Source data for **a, b** are provided at https://doi.org/10.17617/3.AVKYZC, for **c-h** as Source Data files.

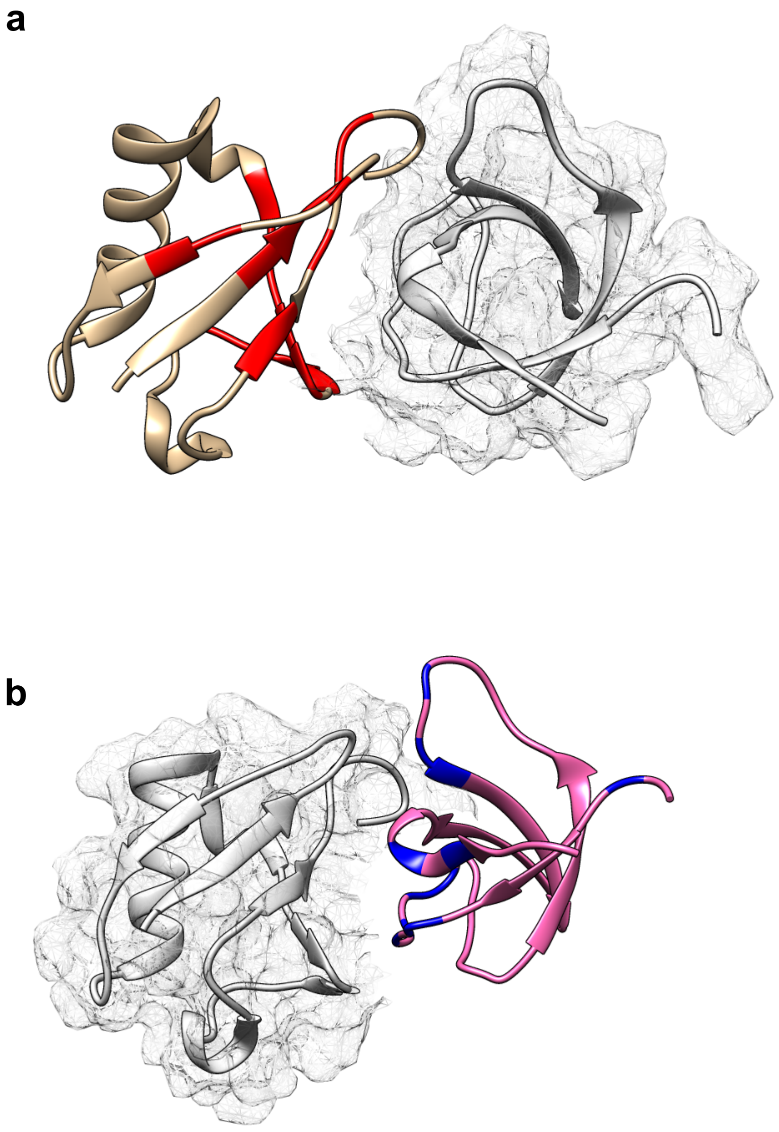

Supplementary Figure 2: **Reporter residues for ubiquitin-SH3c binding.** (a) The 22 ubiquitin residues (shown in red) for which the exchange rate k_{ex} in the presence SH3c is clearly smaller than in free ubiquitin (see text and Supplementary Fig. 3) and, thus, affected by binding. (b) The 12 SH3c residue positions (shown in blue) for which the exchange rate k_{ex} in the presence ubiquitin is clearly smaller than in free SH3c (see text and Supplementary Fig. 4). The position of the partner proteins are shown as grey ribbons inside a transparent solvent-accessible surface representation. The shown structures of the complex corresponds to the PDB file 2K6D.

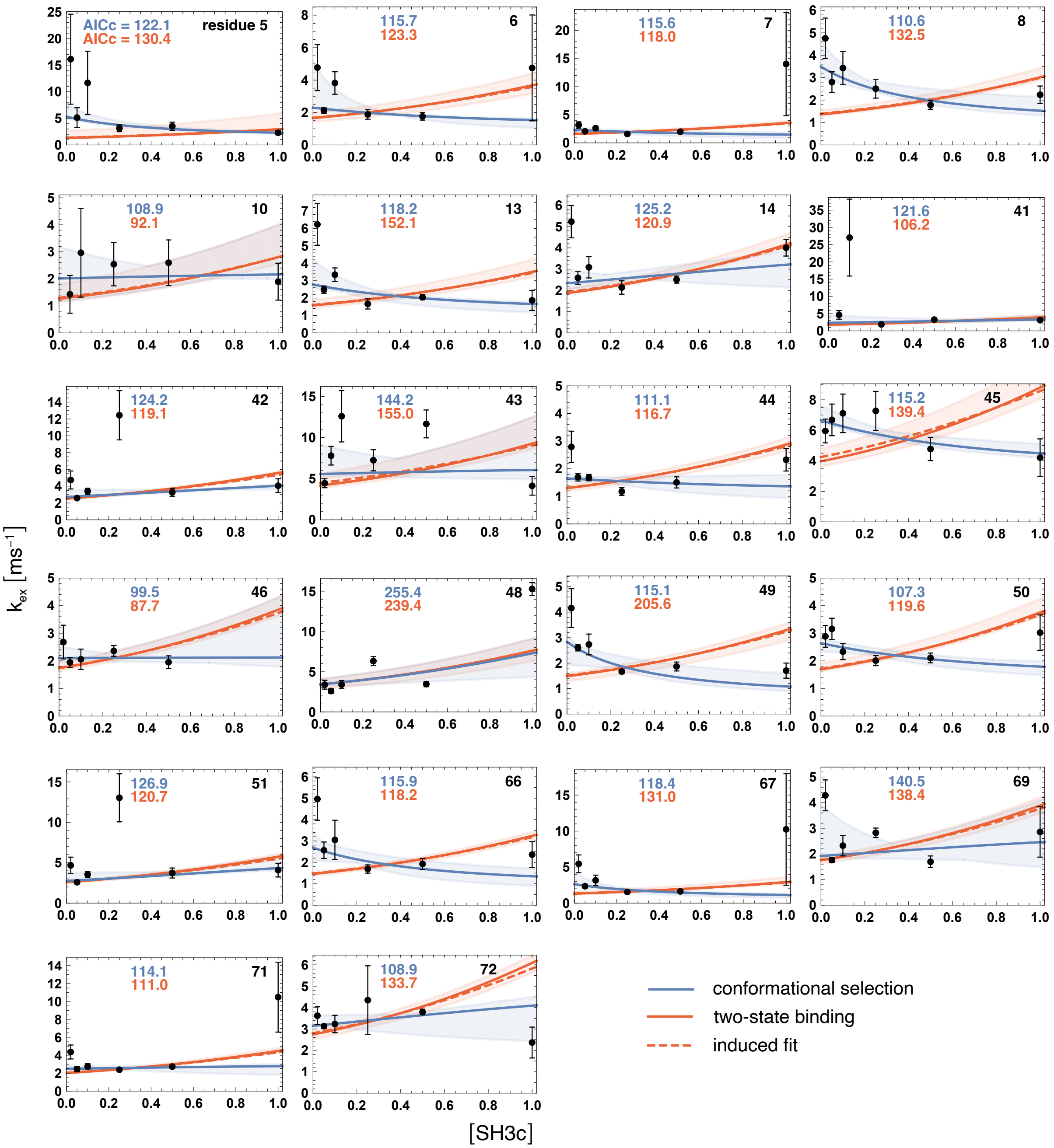

Supplementary Figure 3: **Fits of concentration-dependent exchange rates $k_{\rm ex}$ for ubiquitin**. Fits of $k_{\rm ex}$ from high-power relaxation dispersion experiments at different SH3c concentrations with the two-state binding, conformational-selection, and induced-fit models for all 22 residue positions with $k_{\rm ex}$ values that are clearly smaller than in free ubiquitin and, thus, reflect the exchange between the SH3c-bound and unbound state of ubiquitin. The error bars of data points are standard errors of data fits (Methods). The shaded blue and red regions represent error regions for the two-state binding and conformational-selection model, respectively, from jackknife fits (Supplementary Methods). The finite-size corrected Akaike information criterion (AICc) values for the individual fits of these two models are presented in blue and red. Source data are provided as a Source Data file.

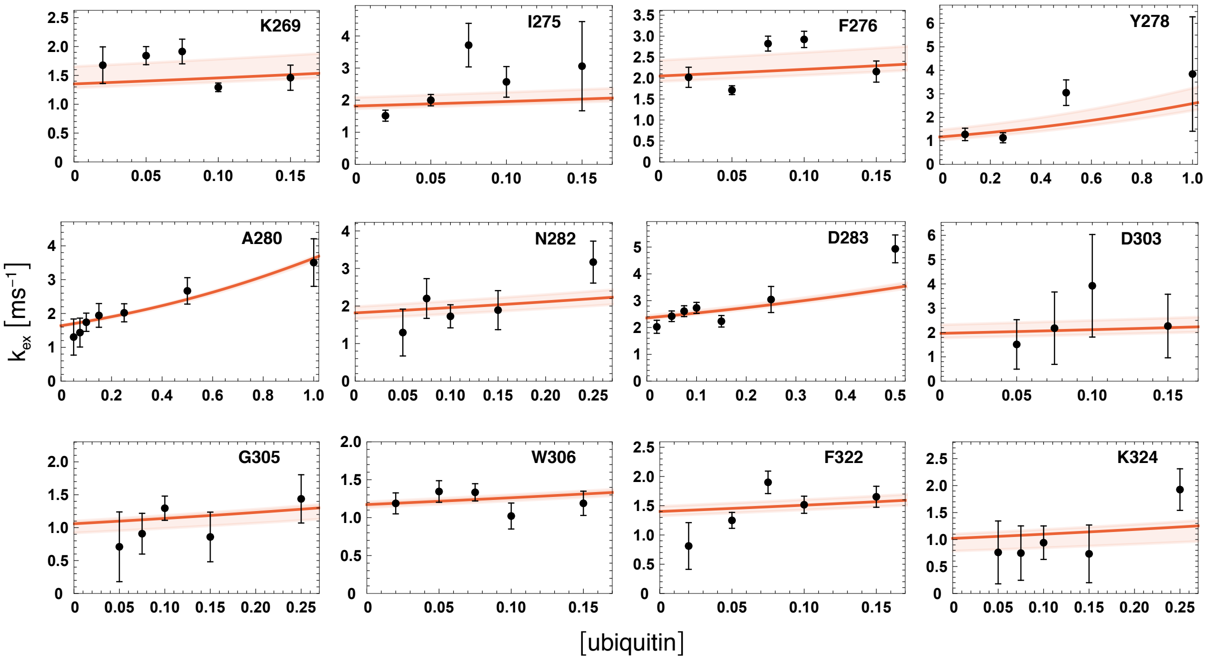

Supplementary Figure 4: **Fits of concentration-dependent exchange rates k_{ex} for SH3c**. Fits of k_{ex} data from relaxation dispersion experiments at different ubiquitin concentrations with the two-state binding model for all 12 residue positions at which k_{ex} is affected by binding to ubiquitin and reflects the exchange between the ubiquitin-bound and unbound state of SH3c. The error bars of data points are standard errors of data fits (Methods). The shaded red regions represent error regions from jackknife fits (Supplementary Methods). Source data are provided as a Source Data file.

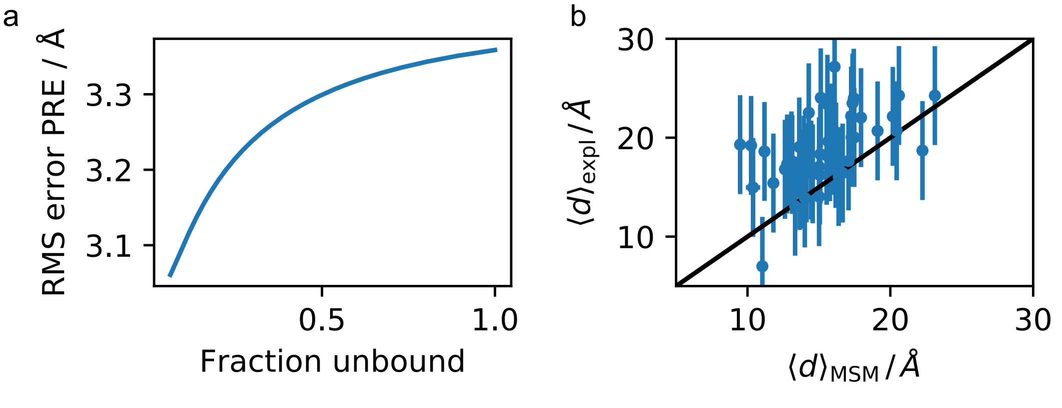

Supplementary Figure 5: **Comparison to previously reported paramagnetic relaxation enhancement (PRE) derived distances**. **a** RMS error between predicted PREs and experimental PREs as a function of the fraction of fully unbound states. **b** Correlation plot of calculated (best fit) and experimental PRE-derived distances (see Supplementary Methods for details). Source data are available at https://github.com/olsson-group/litmus-test-paper.

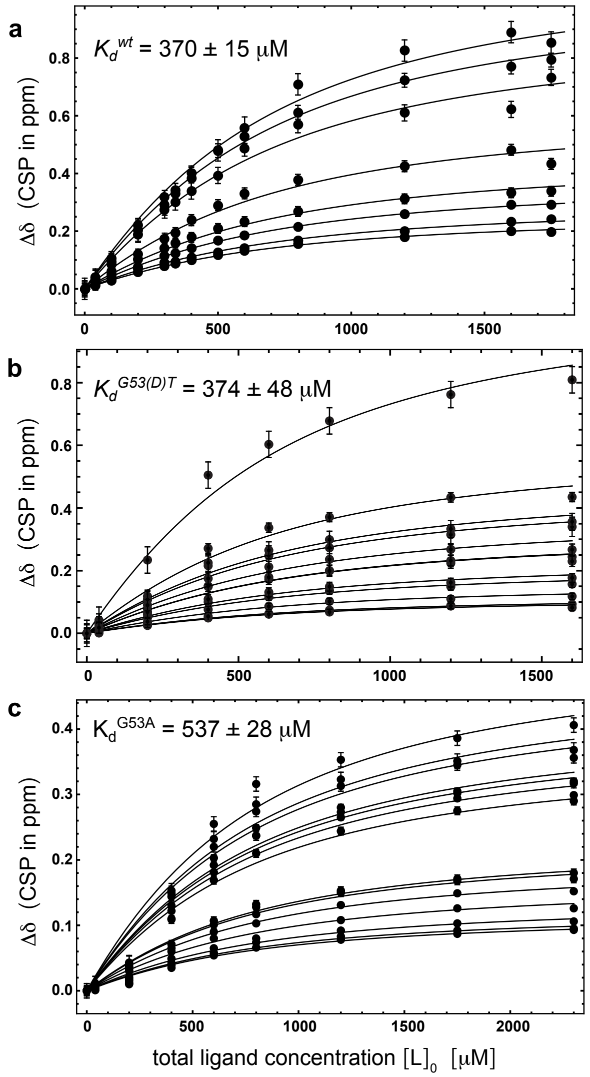

Supplementary Figure 6: **Determination of the dissociation constant K_d from titration observed by NMR chemical shifts**. The chemical shift difference ($\Delta\delta^{\mathrm{N}}_{\mathrm{titr}}$) of various amide proton resonances of free ubiquitin versus ubiquitin in the presence of increasing amount of SH3c are plotted. The global dissociation constant K_d for wild-type ubiquitin, G53(D)T mutant, G53A mutant and SH3c were determined from chemical shift difference. The global ($n = 5$) and residue-specific uncertainties in the datapoints were estimated as described in Supplementary Methods. The largest of the (global or residue-specific) uncertainties are reported. The standard error of the fit parameter is reported. **a** Unlabeled SH3c was titrated into $400\,\mu\mathrm{M}$ ^{15}N labeled wild-type ubiquitin. The titration profiles of representative residues Q2, T14, Q41, L50, E51, T66, L71 and G75 of wild-type ubiquitin are shown. **b** Chemically synthesised G53(D)T mutant of ubiquitin was titrated into $400\,\mu\mathrm{M}$ ^{15}N labeled SH3c. The titration profiles of D263, Y271, A280, L286, K289, N298, K299, E308, E310, R315, G316, F318 and V323 of the SH3c are shown. **c** Unlabeled SH3c was titrated into $400\,\mu\mathrm{M}$ ^{15}N labeled G53A mutant of ubiquitin. The titration profiles of Q2, I3, V5, K11, L15, I23, D32, E34, D39, Q41, D52, K63, E64 and L75 of the G53A mutant of ubiquitin are shown. The K_d values are the best-fit parameters from global fits of titration profiles of a set of residues (using NonlinearModelFit in Mathematica 11.3) of ($n > 100$) datapoints with uncertainities estimated as standard errors of fit. Source data are provided as Source Data files.

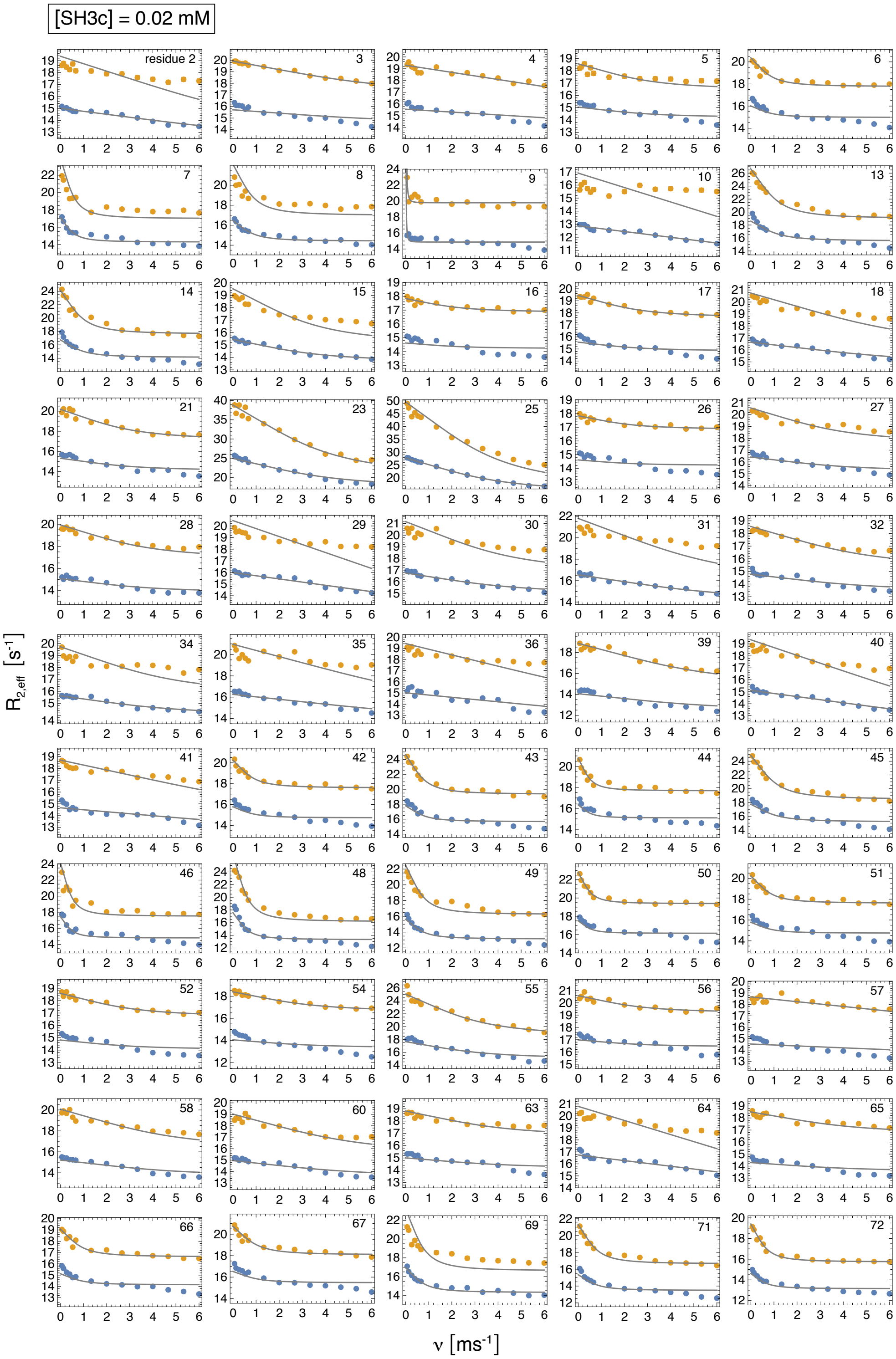

Supplementary Figure 7: Ubiquitin NMR relaxation-dispersion data with fits at the SH3c concentration 0.02 mM. The joint, error-weighted fits of the blue and yellow data sets obtained at the ^{15}N resonance frequencies 60.795 MHz and 96.313 MHz, respectively, tend to be more faithful to the blue data sets because of the smaller errors of these data (see Supplementary Methods for details). The global ($n = 5$) and residue-specific uncertainties in the $R_{2,\mathrm{eff}}$ values were estimated as described in Methods. The largest of the (global or residue-specific) uncertainties are reported (smaller than the plot markers). Source data are provided as Source Data files.

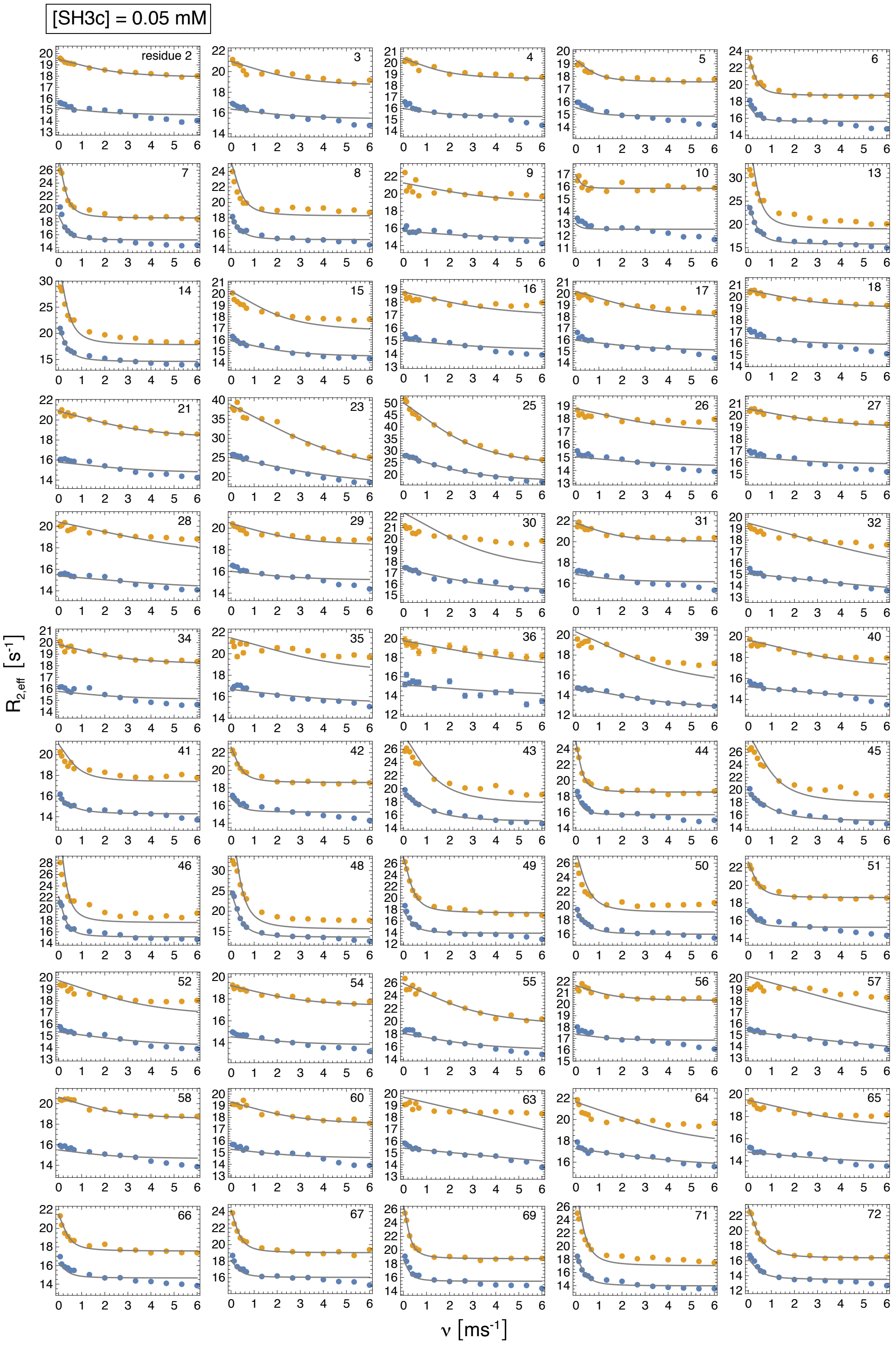

Supplementary Figure 8: Ubiquitin NMR relaxation-dispersion data with fits at the SH3c concentration 0.05 mM. The joint, error-weighted fits of the blue and yellow data sets obtained at the ^{15}N resonance frequencies 60.795 MHz and 96.313 MHz, respectively, tend to be more faithful to the blue data sets because of the smaller errors of these data (see Supplementary Methods for details). The global ($n = 5$) and residue-specific uncertainties in the $R_{2,\mathrm{eff}}$ values were estimated as described in Methods. The largest of the (global or residue-specific) uncertainties are reported (smaller than the plot markers). Source data are provided as Source Data files.

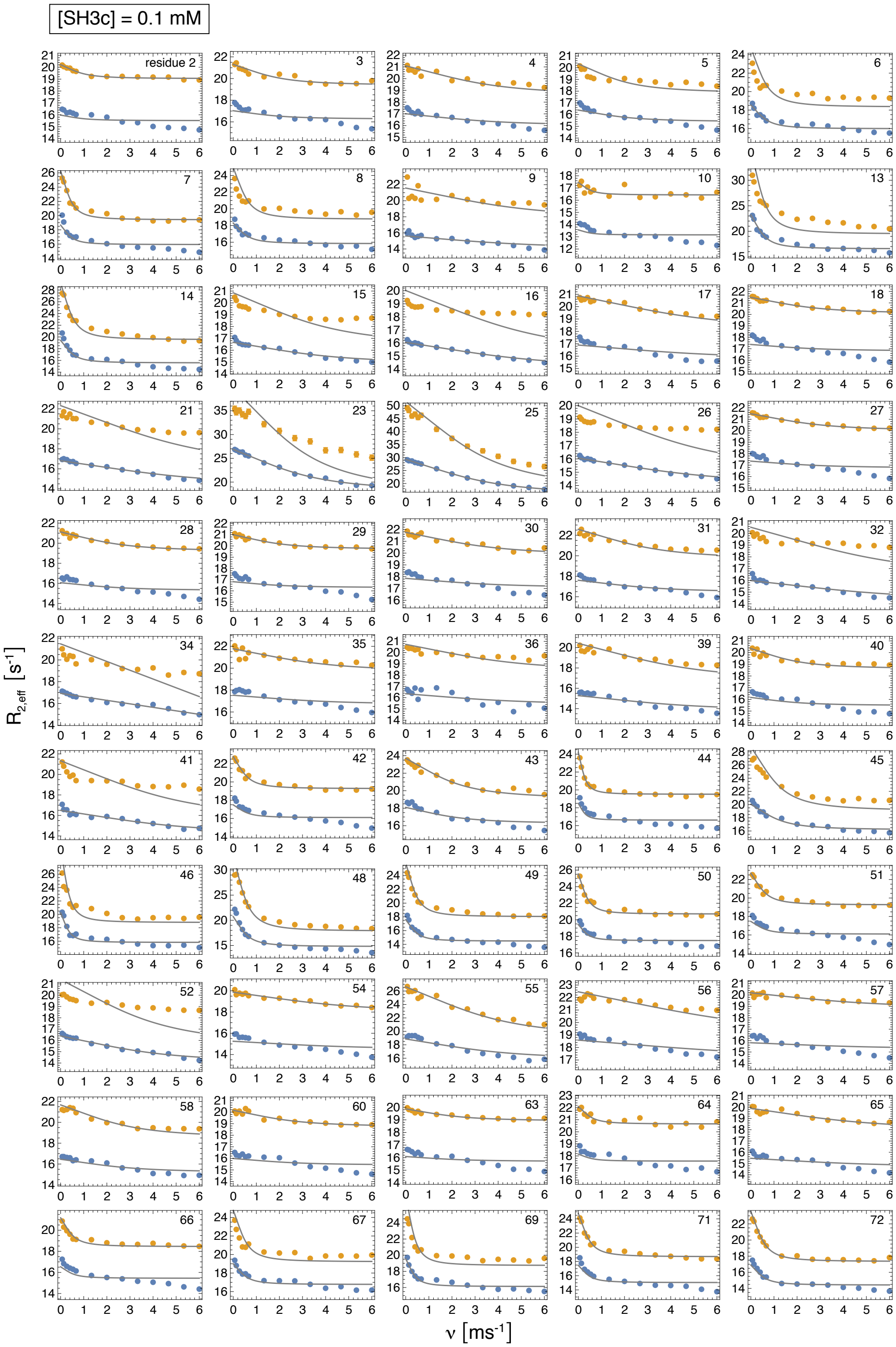

Supplementary Figure 9: Ubiquitin NMR relaxation-dispersion data with fits at the SH3c concentration 0.10 mM. The joint, error-weighted fits of the blue and yellow data sets obtained at the ^{15}N resonance frequencies 60.795 MHz and 96.313 MHz, respectively, tend to be more faithful to the blue data sets because of the smaller errors of these data (see Supplementary Methods for details). The global ($n = 5$) and residue-specific uncertainties in the $R_{2,\mathrm{eff}}$ values were estimated as described in Methods. The largest of the (global or residue-specific) uncertainties are reported (smaller than the plot markers). Source data are provided as Source Data files.

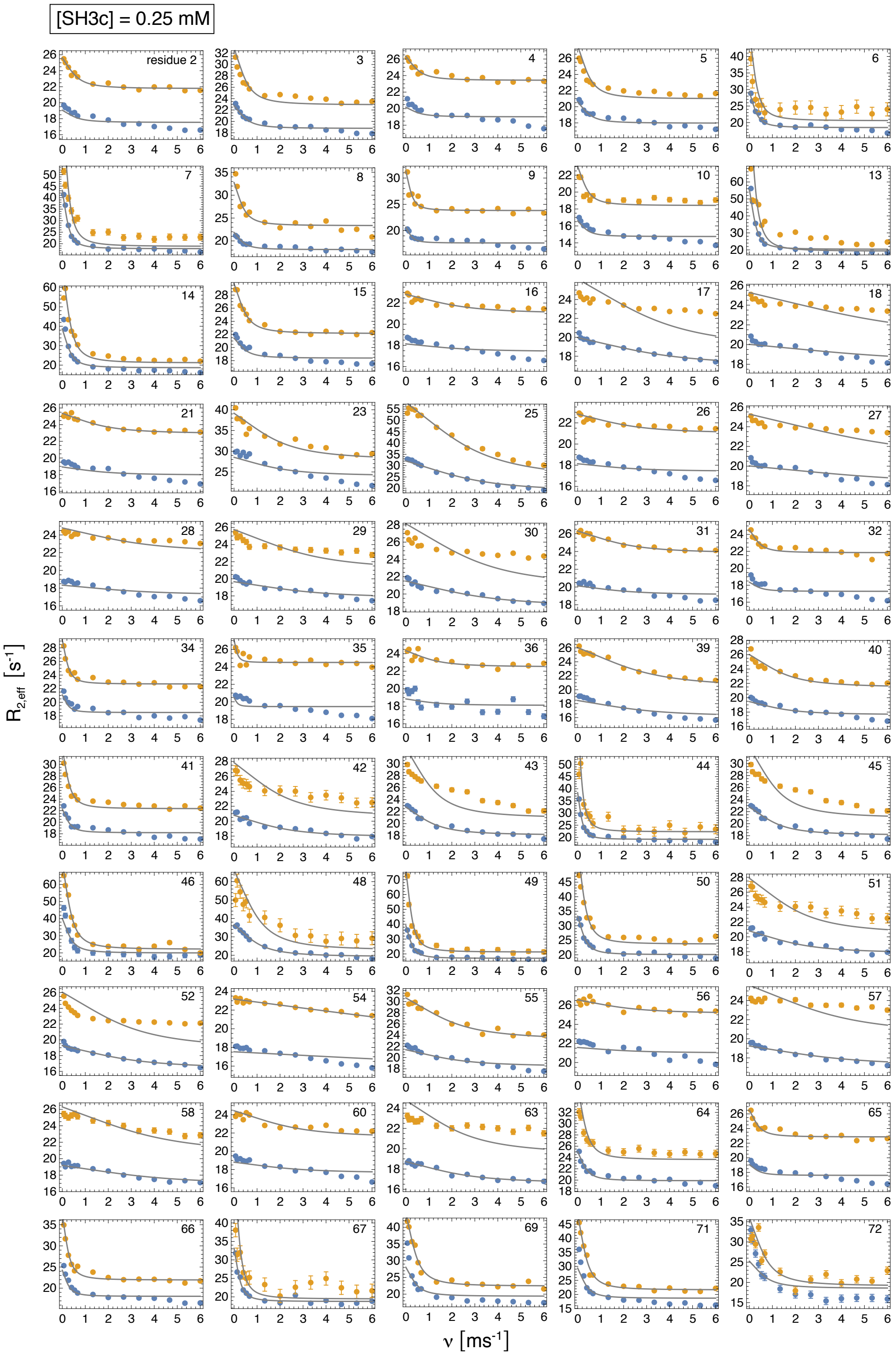

Supplementary Figure 10: Ubiquitin NMR relaxation-dispersion data with fits at the SH3c concentration 0.25 mM. The joint, error-weighted fits of the blue and yellow data sets obtained at the ^{15}N resonance frequencies 60.795 MHz and 96.313 MHz, respectively, tend to be more faithful to the blue data sets because of the smaller errors of these data (see Supplementary Methods for details). The global ($n = 5$) and residue-specific uncertainties in the $R_{2,\mathrm{eff}}$ values were estimated as described in Methods. The largest of the (global or residue-specific) uncertainties are reported. Source data are provided as Source Data files.

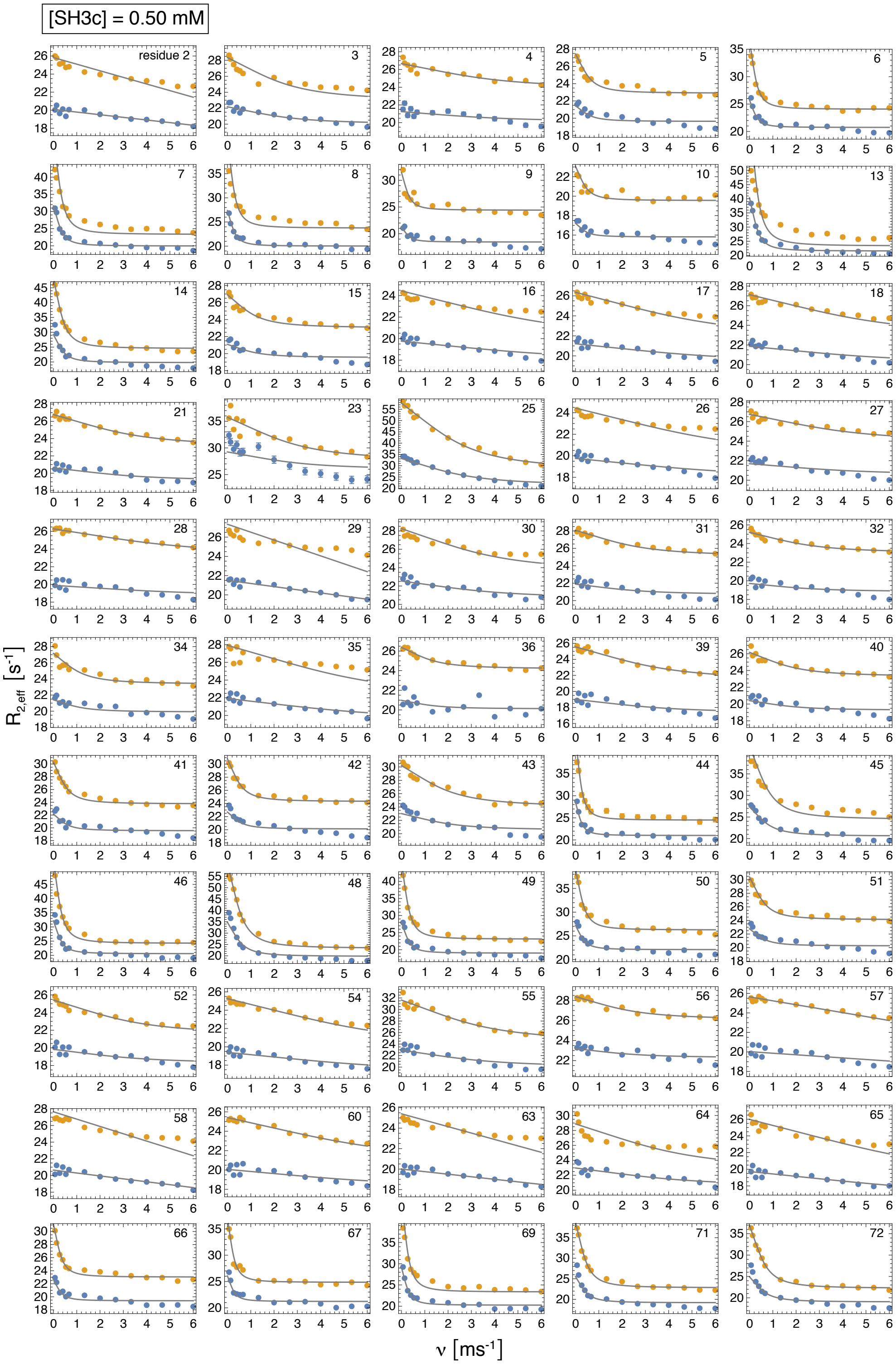

Supplementary Figure 11: Ubiquitin NMR relaxation-dispersion data with fits at the SH3c concentration 0.50 mM. The joint, error-weighted fits of the blue and yellow data sets obtained at the ^{15}N resonance frequencies 60.795 MHz and 96.313 MHz, respectively, tend to be more faithful to the blue data sets because of the smaller errors of these data (see Supplementary Methods for details). The global ($n = 5$) and residue-specific uncertainties in the $R_{2,\mathrm{eff}}$ values were estimated as described in Methods. The largest of the (global or residue-specific) uncertainties are reported. Source data are provided as Source Data files.

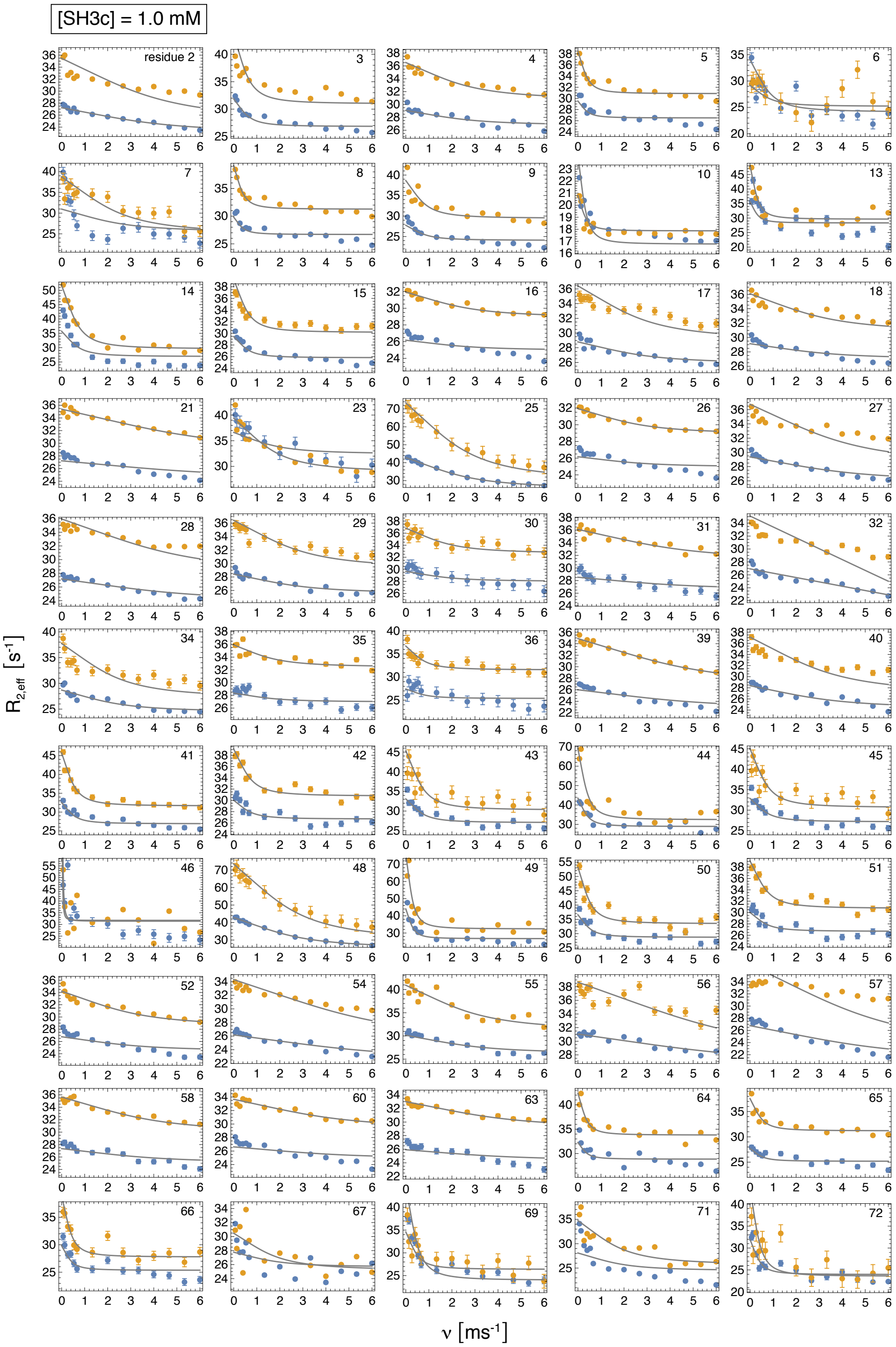

Supplementary Figure 12: Ubiquitin NMR relaxation-dispersion data with fits at the SH3c concentration 1.0 mM. The joint, error-weighted fits of the blue and yellow data sets obtained at the ^{15}N resonance frequencies 60.795 MHz and 96.313 MHz, respectively, tend to be more faithful to the blue data sets because of the smaller errors of these data (see Supplementary Methods for details). The global ($n = 5$) and residue-specific uncertainties in the $R_{2,\mathrm{eff}}$ values were estimated as described in Methods. The largest of the (global or residue-specific) uncertainties are reported. Source data are provided as Source Data files.

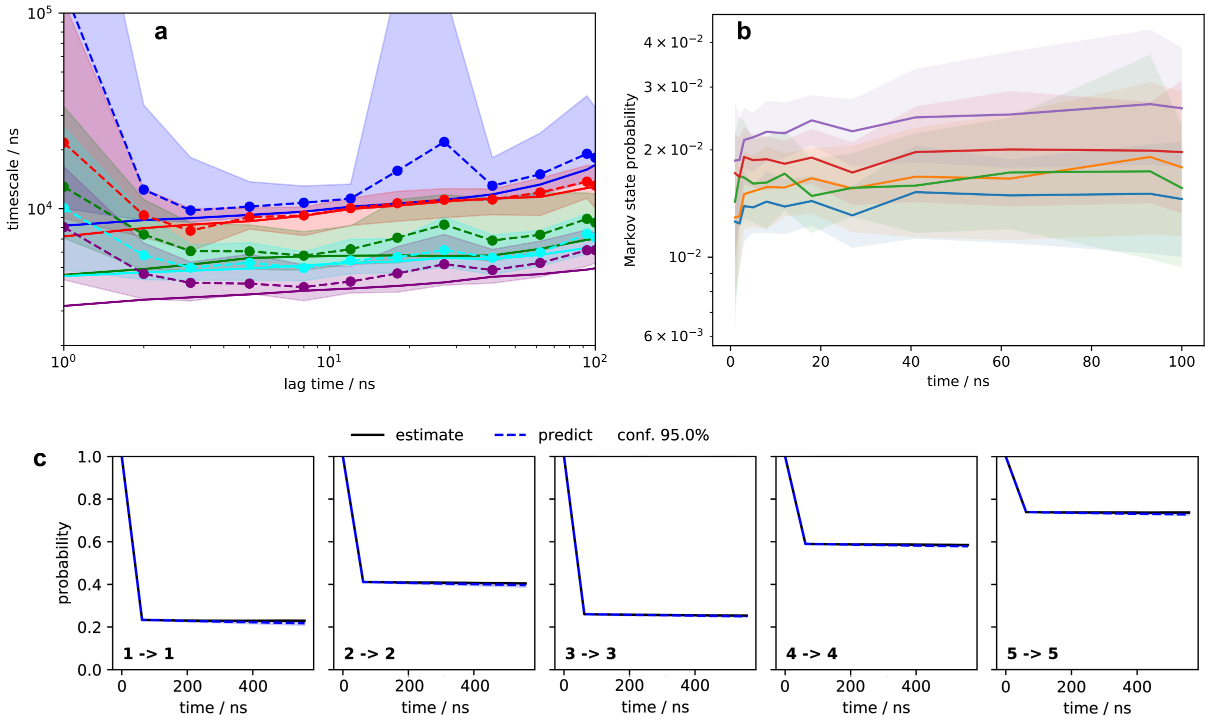

Supplementary Figure 13: **Markov state model (MSM) selection and test**. **a** Implied timescale plot for the ubiquitin-SH3c MSM selection. The implied timescales are the global relaxation timescales predicted by the MSM. **b** State probabilities in MSMs estimated at different timescales. Only the 5 highest populated states are shown. **c** Chapman-Kolmogorov test of the selected model. The model is coarse-grained to five macro-states for this test as there is no clear timescale separation. Plotted values are sample means and 95% confidence intervals as computed using 5000 posterior samples from the Markov model posterior distribution (see Methods for details). Source data are available at https://github.com/olsson-group/litmus-test-paper.

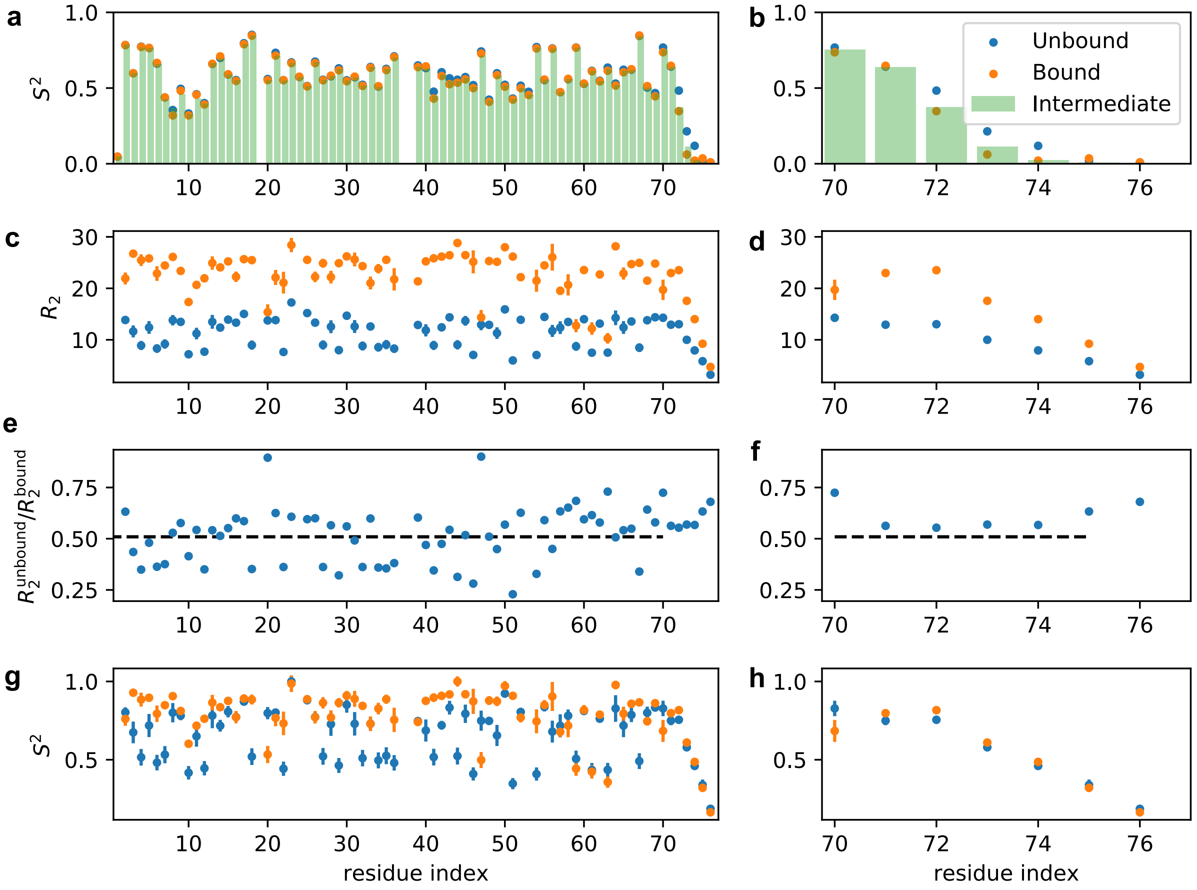

Supplementary Figure 14: **Order parameters and transverse relaxation rates**. **a** Prediction of $^{15}N - {}^{1}H$ order parameters, for bound (orange), unbound (blue), and intermediate state ensembles (green bars), as defined in the Supplementary Methods. **b** The zoomed in view of residues 70 to 76 from **a**. **c** The fitted R_2 values from the high-power relaxation dispersion measurements in 600 MHz spectrometers at 277 K in free (blue) and bound (orange) ubiquitin. **d** The zoomed in view of the residues 70 to 76 from **c**. **e** The ratio of the $R_2^{unbound}/R_2^{bound}$ with respect to the average value (black dashed line). **f** The zoomed in view of the residues 70 to 76 from **e**. **g** The order parameter S^2 was calculated by assuming that the highest R_2 values of residue E24 in both free and bound ubiquitin corresponds to perfect rigidity with $S^2 = 1$. Therefore, the order parameter of individual residues is taken to be $S_i^2 = R_2^i/R_2^{E24}$ for the free (blue) and bound (orange) ubiquitin. **h** The zoomed in view of the residues 70 to 76 from **g**. The uncertainties in R_2 values **c** and **d** are standard errors of fits (using NonlinearModelfFit in Mathematica 11.3) of $(n = 14)$ data points (fits in Supplementary Figs. 7 and 12) with the center as the best-fit value. The propagated errors are shown as error bars in **g** and **h**. Source data for **a, b** are available at https://github.com/olsson-group/litmus-test-paper, for **c-h** as Source Data files.

Supplementary Tables

Supplementary Table 1: Data collection and refinement statistics for G53(D)Thr ubiquitin

**Data collection**	**Statistics**
Space group	$P4_332$
Cell dimensions a, b, c (Å)	$105.479, 105.479, 105.479$
α, β, γ (^{o})	$90, 90, 90$
Resolution (Å)*	$47.17 - 2.60$ $(2.60 - 2.69)$
$R_{int}(\%)$	$9.45(77.93)$
$I/\sigma I$	$41.62(3.91)$
Completeness (%)	$99.9(100.0)$
Redundancy	$51.03(55.26)$
**Refinement**	
Resolution (Å)	2.60
No. reflections	6245
R_{work}/R_{free}	$24.93/29.22$
**No. of atoms**	
Proteins	1181
Cd^{+2}	8
Water	6
**B-factor**	
Proteins	
Chain A	69.73
Chain B	82.62
Cd^{+2}	85
Water	51.67
**R.M.S. deviations**	
Bond lengths (Å)	0.0042
Bond angles (^{o})	1.433
**Ramachandran plot**	
(% of residues in regions)	
most favored	94.41
disallowed	1.4
PDB code	7OOJ

*Values in parenthesis are for highest-resolution shell.

Supplementary Table 2: Ubiquitin k_{ex} values (with errors) in units of ms^{-1} at different SH3c concentrations

residue	0.02 mM	0.05 mM	0.10 mM	0.25 mM	0.50 mM	1.0 mM
2	48.3(46.0)	12.8(2.8)	5.3(0.9)	3.6(0.4)	n.d.	23.0(7.0)
3	33.3(10.6)	14.6(6.3)	10.6(4.0)	3.3(0.6)	14.8(4.6)	3.6(0.8)
4	n.d.	10.3(4.0)	20.1(9.1)	3.4(0.9)	22.0(9.2)	15.0(4.7)
5	16.1(8.4)	5.1(1.9)	11.7(6.0)	3.1(0.6)	3.5(0.8)	2.3(0.4)
6	4.8(1.4)	2.1(0.2)	3.8(0.7)	1.9(0.3)	1.8(0.2)	4.8(3.3)
7	3.2(0.6)	2.1(0.3)	2.6(0.3)	1.6(0.2)	2.0(0.2)	14.0(9.2)
8	4.8(0.9)	2.8(0.5)	3.4(0.7)	2.5(0.4)	1.8(0.2)	2.2(0.4)
9	n.d.	19.9(14.5)	27.0(21.1)	1.5(0.3)	1.8(0.4)	4.7(1.5)
10	n.d.	1.4(0.7)	3.0(1.6)	2.5(0.8)	2.6(0.8)	1.9(0.7)
13	6.2(1.2)	2.5(0.2)	3.3(0.4)	1.7(0.3)	2.0(0.1)	1.9(0.6)
14	5.2(0.8)	2.6(0.3)	3.1(0.5)	2.1(0.3)	2.5(0.2)	4.0(0.4)
15	19.0(3.2)	12.8(3.7)	22.4(7.9)	3.0(0.3)	6.7(1.9)	3.5(0.8)
16	12.1(4.9)	20.0(14.0)	29.6(11.0)	13.6(7.7)	35.7(33.4)	14.9(3.0)
17	13.8(2.2)	16.7(7.4)	30.1(19.0)	21.7(4.8)	31.7(19.7)	16.2(6.0)
18	35.1(20.0)	15.8(7.4)	14.9(3.0)	n.d.	30.5(16.7)	18.4(8.3)
21	15.8(3.7)	19.0(3.2)	31.1(13.1)	10.2(2.6)	19.0(3.0)	33.6(14.8)
23	21.7(3.1)	25.6(5.1)	18.2(1.2)	11.7(3.6)	16.9(6.0)	10.2(2.5)
25	20.8(1.4)	16.3(1.5)	19.9(0.7)	17.4(2.0)	15.1(1.2)	14.9(0.7)
26	12.1(4.9)	20.0(14.0)	29.7(11.2)	13.6(7.7)	35.6(33.2)	15.0(3.1)
27	20.7(9.1)	17.6(7.7)	16.0(3.1)	n.d.	23.7(11.7)	23.4(9.9)
28	19.7(6.4)	29.6(20.3)	14.0(3.8)	19.1(12.3)	29.2(7.5)	27.8(13.2)
29	n.d.	14.8(7.9)	11.3(2.0)	18.8(8.1)	n.d.	16.3(5.1)
30	23.5(8.1)	19.4(4.5)	20.1(7.0)	19.2(4.4)	21.7(9.5)	10.3(4.3)
31	31.7(11.7)	9.7(4.0)	17.9(7.2)	12.2(2.7)	14.8(4.1)	24.5(10.4)
32	25.7(10.9)	40.3(35.6)	32.3(25.1)	2.7(0.5)	14.5(3.3)	n.d.
34	25.1(8.5)	14.5(4.8)	n.d.	1.5(0.2)	6.5(1.8)	13.6(4.5)
35	n.d.	24.2(16.4)	20.8(14.0)	0.9(0.5)	33.2(26.9)	9.6(4.3)
36	n.d.	n.d.	24.4(23.5)	7.3(3.7)	7.7(1.4)	4.5(1.8)
39	28.9(11.2)	22.5(3.3)	25.9(14.5)	16.8(3.4)	20.7(3.9)	27.7(6.0)
40	n.d.	22.7(11.2)	11.0(5.2)	9.3(2.5)	10.6(2.6)	19.4(7.1)
41	n.d.	4.6(1.3)	27.1(11.1)	1.9(0.4)	3.3(0.3)	3.1(0.4)
42	4.7(1.1)	2.6(0.2)	3.4(0.4)	12.4(2.9)	3.3(0.5)	4.1(0.8)
43	4.4(0.5)	7.8(1.1)	12.6(3.1)	7.2(1.3)	11.7(1.7)	4.1(1.1)
44	2.8(0.6)	1.7(0.1)	1.7(0.1)	1.2(0.1)	1.5(0.2)	2.3(0.4)
45	6.0(0.8)	6.7(1.0)	7.1(1.3)	7.3(1.3)	4.8(0.8)	4.2(1.2)

residue	0.02 mM	0.05 mM	0.10 mM	0.25 mM	0.50 mM	1.0 mM
46	2.7(0.6)	1.9(0.2)	2.1(0.4)	2.4(0.2)	2.0(0.2)	n.d.
48	3.4(0.5)	2.6(0.3)	3.4(0.5)	6.3(0.5)	3.5(0.3)	15.3(0.8)
49	4.2(0.8)	2.6(0.1)	2.7(0.4)	1.7(0.1)	1.9(0.2)	1.7(0.3)
50	2.9(0.4)	3.2(0.4)	2.3(0.3)	2.0(0.2)	2.1(0.2)	3.0(0.6)
51	4.7(1.0)	2.6(0.2)	3.5(0.4)	13.0(3.0)	3.7(0.6)	4.1(0.8)
52	16.4(2.8)	20.2(9.5)	22.4(6.3)	17.1(3.3)	19.4(6.6)	17.7(5.7)
54	17.6(2.9)	15.4(4.8)	25.6(9.4)	n.d.	36.1(22.6)	33.9(21.7)
55	17.2(3.0)	17.4(3.1)	22.2(5.8)	12.8(2.2)	19.8(3.8)	17.1(5.3)
56	14.2(4.4)	8.4(3.5)	n.d.	12.8(5.0)	13.7(2.3)	38.5(31.4)
57	n.d.	n.d.	26.4(26.0)	22.1(9.4)	n.d.	31.4(24.6)
58	24.9(10.5)	12.6(2.8)	16.0(4.8)	23.0(7.8)	n.d.	21.5(7.2)
60	27.3(12.6)	15.0(4.7)	14.1(3.9)	14.7(6.9)	36.2(21.3)	24.4(7.4)
63	22.0(13.6)	n.d.	12.5(6.0)	14.6(2.4)	n.d.	23.6(11.4)
64	n.d.	25.1(12.6)	3.2(1.0)	2.3(0.3)	24.9(14.8)	2.1(0.4)
65	22.5(12.8)	23.2(13.9)	25.8(12.7)	2.4(0.5)	n.d.	2.8(0.6)
66	5.0(1.0)	2.6(0.4)	3.1(0.9)	1.7(0.2)	1.9(0.3)	2.4(0.6)
67	5.5(1.2)	2.3(0.3)	3.2(0.7)	1.6(0.2)	1.6(0.2)	10.2(7.8)
69	4.3(0.6)	1.8(0.1)	2.3(0.4)	2.8(0.2)	1.7(0.2)	2.9(1.0)
71	4.4(0.8)	2.5(0.3)	2.8(0.3)	2.4(0.1)	2.7(0.2)	10.5(3.9)
72	3.6(0.4)	3.1(0.1)	3.2(0.4)	4.3(1.6)	3.8(0.1)	2.4(0.7)

”n.d.” (not determined) indicates that the relative fit error is larger than 1. The errors are estimated as standard errors of data fits (Methods). Source data are available as a Source Data file.

Supplementary Table 3: SH3c k_{ex} values (with errors) in units of ms^{-1} at different ubiquitin concentrations

residue	0.02 mM	0.05 mM	0.075 mM	0.10 mM	0.15 mM	0.25 mM	0.50 mM	1.0 mM
D263	n.d.	n.d.	n.d.	0.8(0.8)	n.d.	n.d.	1.4(0.9)	2.4(1.0)
K269	1.7(0.3)	1.8(0.2)	1.9(0.2)	1.3(0.1)	1.5(0.2)			
I275	1.5(0.2)	2.(0.2)	3.7(0.7)	2.6(0.5)	3.1(1.4)			
F276	2.0(0.2)	1.7(0.1)	2.8(0.2)	2.9(0.2)	2.2(0.3)			
Y278	n.d.	n.d.	n.d.	1.3(0.3)	n.d.	1.1(0.2)	3.0(0.5)	3.8(2.4)
A280	n.d.	1.3(0.5)	1.4(0.4)	1.7(0.3)	1.9(0.4)	2.0(0.3)	2.7(0.4)	3.5(0.7)
N282	n.d.	1.3(0.6)	2.2(0.5)	1.7(0.3)	1.9(0.5)	3.2(0.6)		
D283	2.0(0.2)	2.4(0.2)	2.6(0.2)	2.7(0.2)	2.2(0.2)	3.9(0.5)	4.9(0.5)	
D284	n.d.	n.d.	3.0(1.4)	3.6(1.2)	2.2(1.2)			
L286	n.d.	n.d.	n.d.	n.d.	n.d.	2.2(0.9)	1.5(0.9)	7.2(2.9)
K289	n.d.	n.d.	n.d.	n.d.	n.d.	0.9(0.8)	2.6(1.2)	n.d.
D292	n.d.	1.0(0.9)	n.d.	1.4(0.6)	n.d.	n.d.	n.d.	n.d.
D303	n.d.	1.5(1.0)	2.2(1.5)	3.9(2.1)	2.3(1.3)	n.d.	n.d.	
G305	n.d.	0.7(0.5)	0.9(0.3)	1.3(0.2)	0.9(0.4)	1.4(0.4)		
W306	1.2(0.1)	1.3(0.1)	1.3(0.1)	1.0(0.2)	1.2(0.2)			
R315	n.d.	n.d.	n.d.	n.d.	n.d.	n.d.	2.7(1.5)	2.9(1.0)
F318	n.d.	n.d.	n.d.	n.d.	n.d.	n.d.	4.0(2.4)	n.d.
D320	1.5(0.3)	2.2(0.2)	3.7(1.1)					
F322	0.8(0.4)	1.2(0.1)	1.9(0.2)	1.5(0.1)	1.7(0.2)			
K324	n.d.	0.8(0.6)	0.7(0.5)	0.9(0.3)	0.7(0.5)	1.9(0.4)		

"n.d." (not determined) indicates that the relative fit error is larger than 1. The errors are estimated as standard errors of data fits (Methods). Source data are available as a Source Data file.

Supplementary Table 4: Residue pairs used as input for TICA based dimensionality reduction. All pair-wise combinations of elements in each column (within each subtable) is used as a distance feature with the shortest heavy atom distance.

residue ubiquitin	residue SH3c
F4	W306
L8	P319
I44	F322
H68	F276
V70	F378
L71	
L73	

residue ubiquitin	residue SH3c
R74	D283
	D300
	D298

Supplementary Table 5: Equilibrium probability p_i, binding committor p_{bind}, net reactive flux (computed as described in Ref. 74, in units of the inverse MSM lag-time), and probability p_{compact} of the compact C-terminal conformation in the coarse-grained macrostates of the MSM. State 14 is fully bound and states P1 and P2 are unbound. Sub- and super-scripts indicate the 95% confidence intervals. Source data are available as a Source Data file.

state	p_i (%)	p_{bind}	net reactive flux	p_{compact}
P1	$0.0758^{0.135}_{0.0403}$	0.0	$2.5 \cdot 10^{-6}$	$1.0^{1.0}_{1.0}$
P2	$0.222^{0.318}_{0.151}$	0.0	$8.3 \cdot 10^{-6}$	$0.0^{0.0}_{0.0}$
0	$0.264^{1.37}_{0.036}$	0.57	$8.1 \cdot 10^{-9}$	$0.0^{0.0}_{0.0}$
1	$0.272^{0.679}_{0.0956}$	0.93	$2 \cdot 10^{-8}$	$0.0^{0.0}_{0.0}$
2	$0.581^{1.4}_{0.204}$	0.58	$1.4 \cdot 10^{-7}$	$0.0^{0.0}_{0.0}$
3	$0.565^{1.48}_{0.211}$	0.52	$1.1 \cdot 10^{-7}$	$0.0^{0.0}_{0.0}$
4	$0.541^{1.4}_{0.135}$	0.92	$4.4 \cdot 10^{-9}$	$0.0^{0.0}_{0.0}$
B	$2.56^{3.61}_{1.74}$	0.59	$1.1 \cdot 10^{-6}$	$0.002^{0.005}_{0.000632}$
6	$1.02^{2.31}_{0.398}$	0.66	$7.2 \cdot 10^{-8}$	$0.00956^{0.0384}_{0.00129}$
7	$4.33^{7.26}_{2.72}$	0.51	$1.3 \cdot 10^{-6}$	$0.0575^{0.106}_{0.0264}$
A	$6.08^{8.24}_{4.3}$	0.57	$1.6 \cdot 10^{-6}$	$0.00473^{0.0144}_{0.00126}$
9	$1.12^{2.37}_{0.372}$	0.8	$7.1 \cdot 10^{-8}$	$0.0^{0.0}_{0.0}$
10	$2.11^{3.03}_{1.42}$	0.8	$8.7 \cdot 10^{-7}$	$0.0^{0.0}_{0.0}$
E	$8.67^{11.2}_{6.64}$	0.85	$3.2 \cdot 10^{-6}$	$0.0674^{0.0946}_{0.0451}$
D	$9.17^{13.2}_{6.13}$	0.75	$1.4 \cdot 10^{-6}$	$0.223^{0.292}_{0.162}$
C	$24.0^{28.7}_{19.8}$	0.67	$6.5 \cdot 10^{-6}$	$0.0271^{0.0411}_{0.0177}$
F	$38.4^{45.6}_{31.7}$	1.0	$1.1 \cdot 10^{-5}$	$0.0583^{0.0785}_{0.0425}$
